# Skin-like mechanoresponsive self-healing ionic elastomer from supramolecular zwitterionic network

**DOI:** 10.1038/s41467-021-24382-4

**Published:** 2021-07-02

**Authors:** Wei Zhang, Baohu Wu, Shengtong Sun, Peiyi Wu

**Affiliations:** 1 State Key Laboratory for Modification of Chemical Fibers and Polymer Materials, College of Chemistry, Chemical Engineering and Biotechnology and Center for Advanced Low-dimension Materials, Donghua University, Shanghai, China; 2 Jülich Centre for Neutron Science (JCNS) at Heinz Maier-Leibnitz Zentrum (MLZ) Forschungszentrum Jülich, Garching, Germany

**Keywords:** Gels and hydrogels, Electronic devices, Gels and hydrogels

## Abstract

Stretchable ionic skins are intriguing in mimicking the versatile sensations of natural skins. However, for their applications in advanced electronics, good elastic recovery, self-healing, and more importantly, skin-like nonlinear mechanoresponse (strain-stiffening) are essential but can be rarely met in one material. Here we demonstrate a robust proton-conductive ionic skin design via introducing an entropy-driven supramolecular zwitterionic reorganizable network to the hydrogen-bonded polycarboxylic acid network. The design allows two dynamic networks with distinct interacting strength to sequentially debond with stretch, and the conflict among elasticity, self-healing, and strain-stiffening can be thus defeated. The representative polyacrylic acid/betaine elastomer exhibits high stretchability (1600% elongation), immense strain-stiffening (24-fold modulus enhancement), ~100% self-healing, excellent elasticity (97.9 ± 1.1% recovery ratio, <14% hysteresis), high transparency (99.7 ± 0.1%), moisture-preserving, anti-freezing (elastic at −40 °C), water reprocessibility, as well as easy-to-peel adhesion. The combined advantages make the present ionic elastomer very promising in wearable iontronic sensors for human-machine interfacing.

## Abstract (web summary)

Ionic skins are of interest for a range of electronic, sensing and interfacing applications but often have trade-offs in properties. Here, the authors report on the creation of a dual network ionic skin using a supramolecular zwitterionic competing network to produce a strain-stiffening, self-healing adhesive sensor.

## Introduction

Skin, covering the body of a vertebrate animal, plays a key role in protecting the inner soft tissues and responding to various external stimuli in shaping our interactions with the world^1^. Inspired by the ion-conducting nature and sensory functions of the skin, artificial ionic skins based on stretchable ionic conductors like hydrogels, ionogels, and ion-conducting elastomers have received considerable attention resulting in a series of temperature, pressure, and strain sensors^2–6^. Similar to many biological issues, skin is also self-repairable and self-protective. As is known, human skin can autonomously heal from wounds to restore its mechanical and electrical properties^7^. More intriguingly, unlike the vast majority of elastomeric materials, skin shows a nonlinear J-shaped stress–strain mechanoresponse (strain-stiffening); that is, skin is soft to touch yet rapidly stiffens to prevent injury due to a sharp increase of elastic modulus at large strains^8,9^. Such a unique mechanoresponsive behavior represents one of nature’s key defense mechanisms, which is attributed to its composite structure comprising stiff collagen fibers to resist deformation and interwoven elastin network to ensure elastic recoil. In sharp contrast, although these mechanical properties are also highly desirable for the compliance, healability, and self-protection of skin-like wearable electronic devices, artificial ionic skins that mimic the all-round sensation, self-healing, and strain-stiffening properties of natural skins are still rare.

While highly stretchable and self-healable ionic skins have been frequently reported^10–14^, most of the synthetic ionic conductors are strain-softening. There is often a conflict among elasticity, self-healability, and strain-stiffening for stretchable ionic conductors. In traditional elastomers, good elasticity relies on strong covalently bonded crosslinks, which allow the material to fully recover its original state driven by entropic gain^15^. In contrast, self-healing generally occurs through the reorganization of the intrinsic elastic network by regenerating dynamic non-covalent bonds^7^. Stretching such a dynamic network is usually accompanied by crosslinking density reduction and stress relaxation, leading to the remarkable attenuation of material modulus as well as poor elastic recovery from large deformations^14,16–19^. On the other hand, strain-stiffening materials normally involve two distinct networks with different rigidities that unfold progressively for synergizing softness and firmness^20–23^. For instance, bottlebrush elastomers could replicate the strain-stiffening characteristics of biological tissues by unfolding flexible strands at lower forces followed by stretching rigid backbone at higher forces^9,21–24^. In other reported elastomers, integrating permanent chemical crosslinks or crystalline domains with weak intermolecular crosslinks may also lead to strain-induced modulus increase^25–27^. Therefore, one major challenge to synthesize elastic, self-healing yet strain-stiffening ionic conductors arises in designing multiscale polymer networks, which concurrently possess dynamic yet strong crosslinks as well as supramolecular weak bonding to mimic the respective roles of stiff collagen fiber and soft elastin matrix in natural skin. Tian et al. recently developed a hybrid elastic hydrogel featuring cell-like starch granules embedded in a crosslinked polyacrylamide matrix, which displays both tissue-like strain-stiffening and self-healing behaviors through dynamic hydrogen bonding and granular interactions; however, in their system, chemical crosslinks are still existing, and thus only ~90% healing efficiency was observed^28^. To the best of our knowledge, there is thus far no report on stretchable ionic conductors with combined good elasticity, full self-healability, and unique strain-stiffening properties.

Herein, we report the design and preparation of a series of highly elastic, transparent, self-healable, and strain-stiffening proton-conductive ionic skins by introducing an entropy-driven supramolecular zwitterionic competing network to the hydrogen-bonded (H-bonded) polycarboxylic acid chain network. Different from hydrogels and ionogels that rely on the use of large amounts of solvents, only the equilibrium moisture content of water is existing in the present ionic elastomers. This feature makes the intermolecular dimeric H-bonds strong enough to crosslink polycarboxylic acid chains at ambient conditions, yet become dynamic as immersed in high humidities to allow for full self-healing. Importantly, the zwitterionic network consisting of weakly complexed zwitterions contributes to the initial softness of the ionic elastomer, which subsequently fragments during stretch resulting in an immensely stiffened H-bonded polycarboxylic acid network. Such a sequential debonding of two competing dynamic networks, as well as the rapid entropy-driven reorganization of zwitterions, leads to ultrahigh stretchability (1600% elongation), apparent strain-stiffening (24 times enhancement of differential modulus), full self-healability (almost 100% efficiency), and excellent elastic recovery (97.9 ± 1.1% recovery ratio, <14% hysteresis), in the case of the representative polyacrylic acid (PAA)/betaine elastomer. The presence of zwitterions also renders the ionic elastomers with moisture-preserving and anti-freezing advantages, allowing the elastomers to steadily conduct protons even in harsh conditions. In addition, the resulting ionic skins are highly adhesive to readily adhere on various substrates and human skins, yet easily peeled off due to the inherent strain-stiffening effect. More interestingly, the ionic skin can be recycled by quickly dissolving in water and recasting in air. As skin-like sensors, the ionic elastomers demonstrate timely response to strain and temperature changes, and can be further integrated with elastic conductive fabrics as an iontronic smart sensor to perceive pressure changes, demonstrating its great potential in wearable electronics.

## Results

### Molecular design of mechanoresponsive PAA/zwitterion elastomers

**Fig. 1:**
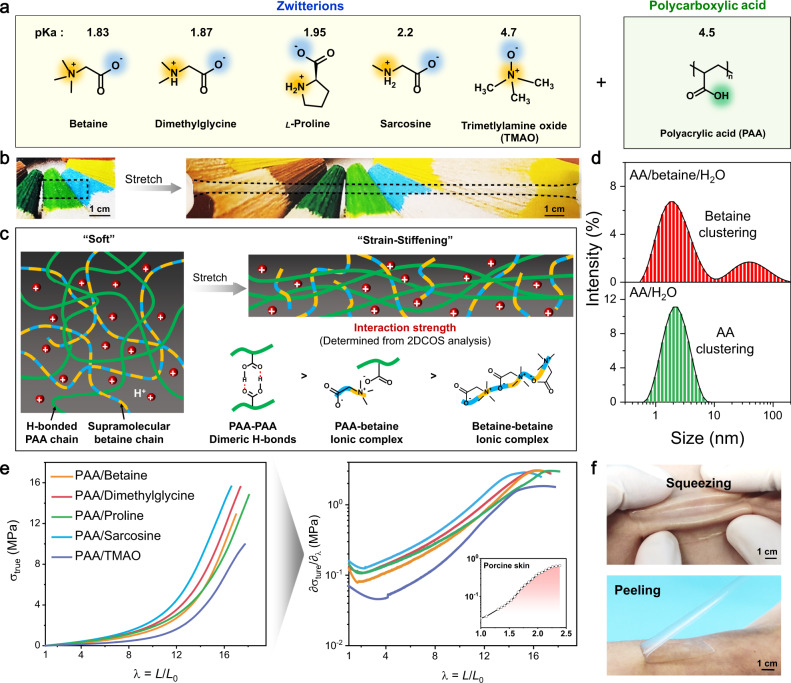
Molecular design and skin-like nonlinear mechanoresponse of PAA/zwitterion elastomers. **a** Molecular structures and p*K*_a_ values of zwitterions and polyacrylic acid (PAA) for the preparation of proton-conductive ionic skins. **b** A transparent PAA/betaine elastomer film was stretched 12 times. **c** Schematic structure of PAA/betaine elastomer before and after stretch and the order of interaction strength among the three main interacting pairs as determined from the following 2DCOS analysis. Water molecules are not shown for clarity. As stretched, the supramolecular betaine chain network gradually fragments, leading to the immense stiffening of the elastomer arising from strongly H-bonded and extended PAA chains. **d** DLS size distribution curves of AA/H_2_O (molar ratio, 1:2.5) and AA/betaine/H_2_O (molar ratio, 1:1:2.5) solutions. Note that the sub-nanometer sizes may be less precisely determined in the single large scattering angle DLS measurement due to the polydispersity effect^51^. **e** True stress and corresponding differential modulus curves of as-prepared PAA/zwitterion elastomers as a function of elongation (stretching rate: 100 mm min^−1^). The inset is the typical differential modulus–elongation curve of porcine skin reproduced from literature^9^. **f** PAA/betaine elastomer is mechanically compliant and adhesive to human skin, yet easily peeled off due to the inherent strain-stiffening effect.

Zwitterions, also called internal salts or dipolar ions, are small molecules containing an equal number of cationic and anionic functional groups in the same structure with an overall neutral charge, which perform important biological functions ranging from osmotic pressure regulation to the modification of cell surface properties^29,30^. As shown in Fig. 1a, we selected five representative zwitterions, including betaine, dimethylglycine, l-proline, sarcosine, and trimethylamine oxide (TMAO), to participate in the polymerization of acrylic acid (AA) for synthesizing a series of proton-conductive elastomers. The preparation process is rather simple by mixing AA, zwitterion, and water in the molar ratio of 1:1:2.5 with Irgacure 2959 as the photoinitiator, followed by ultraviolet (UV)-induced polymerization of AA for 30 min. It is noted that the addition of a specified amount of water has two main roles: (1) the resulting water content in the as-prepared ionic elastomers is almost equal to the equilibrium moisture content at a relative humidity (RH) of 60%, which avoids drastic dehydration or hydration in air. (2) The presence of a proper amount of water would significantly weaken the direct contact ion pairs of zwitterions^31^, yet the strong dimeric H-bonds between PAA chains are not largely influenced, leading to a plasticized elastic network. As a result, all the synthesized elastomers are highly stretchable and transparent (Fig. 1b, Supplementary Fig. 1, and Movie 1). For example, the optical transmittance of PAA/betaine elastomer in the visible region can reach 99.7 ± 0.1% (Supplementary Fig. 2).

We highlight that nearly all the acid dissociation constants (p*K*_a_) of zwitterions are lower than that of PAA in their bulk aqueous solutions (Fig. 1a), suggesting that zwitterions do not significantly deprotonate the carboxylic acid groups of PAA. Therefore, in the PAA-zwitterion system, three main interactions for chain crosslinking are assumed to exist with reduced bonding strength: COOH dimeric H-bonds, polymer–zwitterion complex, and zwitterion–zwitterion complex, as illustrated in Fig. 1c (the order will be confirmed in the following two-dimensional correlation spectroscopy (2DCOS) analysis; note that the latter two interactions are both water-involved, but for the convenience of discussion, water molecules are not shown in the scheme). Both previous Raman and simulation results implied that high-concentration zwitterions like betaine and TMAO in water would form chain-like aggregation due to the electrostatic interactions between negatively charged carboxylate/oxygen and positively charged nitrogen^32–34^. To further evidence the presence of  the supramolecular zwitterion network, we performed the dynamic light scattering (DLS) analyses of AA/betaine/H_2_O reaction precursor, AA/H_2_O, and saturated betaine solutions. As shown in Fig. 1d, there are two peaks in the reaction precursor; the size at 2 nm is ascribed to AA clustering via H-bonds, and the size at 37.8 nm should arise from betaine clustering, which is much smaller than that in saturated betaine solution (centered at 615 nm, Supplementary Fig. 3) due to AA-induced pH decrease. Interestingly, the interactions between AA and betaine also contribute to their cosolvency in water (Supplementary Fig. 4), which is reminiscent of deep eutectic solvents with enhanced solubility due to strong hydrogen bonding^35^.

All the above results support a hypothesis that there might be two kinds of chain networks in the PAA/zwitterion elastomer (Fig. 1c): taking PAA/betaine, for example, one is the covalent PAA chains self-crosslinked by strong dimeric H-bonds, and the other is the fugitive supramolecular betaine chains formed by weak ionic complexes. Obviously, these two networks are not independent, but associated via moderate-strength PAA–betaine ionic complexes, which share the same carboxylate groups. In the original state, the weakly bonded betaine network and random-coil PAA chains contribute to the softness of the elastomer, while as stretched, the fragile betaine chains readily fragment resulting in a stiffened network dominated by strongly H-bonded and extended PAA chains. It is noted that such a covalent-supramolecular dual-network design is distinct from previously reported polyzwitterion-based elastomers^10,11,14,36–39^, which are strain-softening due to stretch-induced monotonic disassociation of chain crosslinks.

Judging from the true stress- and corresponding differential modulus–elongation curves (Fig. 1e), all the PAA/zwitterion elastomers exhibit intense strain-stiffening behaviors similar to the characterisitics of porcine skin^9^. The almost identical tensile curves of the five PAA/zwitterion elastomers in three batches each indicate that such unique mechanoresponse is fully reproducible (Supplementary Fig. 5). The maximum elongations of the as-prepared PAA-based elastomers are ca. 1700%. It is noted that the differential modulus (∂*σ*_true_/∂*λ*) curves of PAA/zwitterion elastomers as a function of deformation ratio (*λ*) show a unique sigmoid shape, which contrasts with the steady increase in stiffness displayed by traditional synthetic elastomers^9^, yet coincides with natural skin’s deformation response more precisely. In the case of the same molar ratio, the type of zwitterions presents relatively small differences in the stiffening trend, yet the initial moduli are affected to a certain content. As shown in Supplementary Fig. 6, the initial modulus of PAA/TMAO is the lowest, which is ascribed to the highest deprotonation degree of PAA (Supplementary Fig. 7). The initial moduli of the other four PAA/zwitterion elastomers slightly increase in the order of betaine, dimethylglycine, proline, and sarcosine, arising from mainly the increasing relative amount of H-bonded PAA as elucidated by pH and attenuated total reflection-Fourier transform infrared (ATR-FTIR) spectral comparison (Supplementary Fig. 7). In the case of PAA/betaine, the differential modulus first slightly decreases from 0.12 to 0.08 MPa in the strain range of 0–70%, and then drastically increases to 3.0 MPa at the maximum 1600% strain, corresponding to ~24 times stiffness enhancement. Decreasing the amount of betaine in the PAA/betaine elastomer significantly weakens the strain-stiffening effect as well as stretchability (Supplementary Fig. 8), verifying the importance of dual-network design. Control samples by replacing betaine with other ionic salts/acids such as lithium bromide (LiBr) and CH_3_COOH show much weakened tensile strength and stretchability as well as diminished strain-stiffening effect (Supplementary Fig. 9), further consolidating the role of the supramolecular betaine network. Moreover, as observed in Fig. 1f, the PAA/betaine elastomer is both mechanically compliant and adhesive to adapt to human skin curvatures, yet easily peeled off due to the intense strain-stiffening effect (Supplementary Movie 1). The cytotoxicity tests with both HeLa and HepG2 cells prove the good biocompatibility of PAA/betaine elastomer that is suitable for on-skin applications (Supplementary Fig. 10).

### Internal interactions in PAA/betaine elastomer

**Fig. 2:**
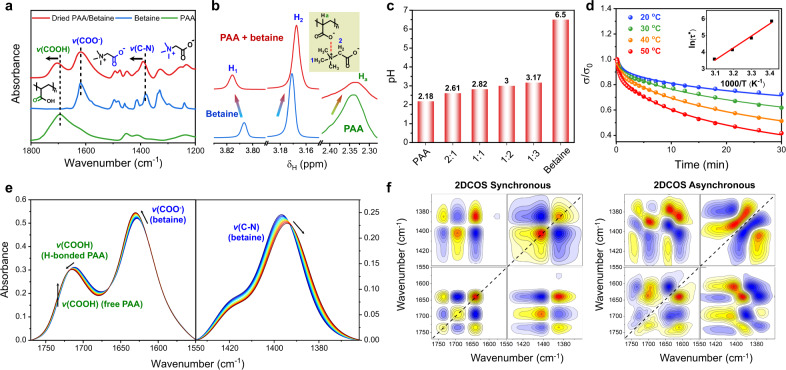
Internal interactions of PAA/betaine elastomer. **a** FTIR spectra and assignments of PAA, betaine, and dried PAA/betaine. **b** ^1^H NMR spectra of PAA, betaine, and PAA/betaine in D_2_O. The total solute concentrations are all 0.1 M. **c** pH values of 0.1 M PAA/betaine solutions with different AA:betaine molar ratios. **d** Iso-strain–stress relaxation curves at different temperatures. The inset is the corresponding fitted result according to Arrhenius equation. **e** Temperature-variable FTIR spectra of PAA/betaine elastomer upon heating from 20 to 70 °C in the regions of *v*(C = O) and *v*(C–N) (interval: 5 °C). **f** 2DCOS synchronous and asynchronous spectra generated from (**e**). In 2DCOS spectra, the warm colors (red and yellow) represent positive intensities, while cold colors (blue) represent negative intensities.

To validate the internal interactions in the representative PAA/betaine elastomer, ATR-FTIR spectra of PAA, betaine, and dried PAA/betaine were compared in Fig. 2a. The FTIR spectrum of betaine exhibits two clear peaks at 1618 and 1380 cm^−1^ for *v*(COO^−^) and *v*(C–N), respectively^40^, and the peak at 1695 cm^−1^ corresponds to *v*(C = O) of PAA with dimeric H-bonds^41^. As for dried PAA/betaine, both the *v*(C = O) of PAA and *v*(C–N) of betaine shift to higher wavenumbers, while *v*(COO^−^) of betaine is almost unchanged, which reveals the ionic complexation between PAA and betaine. Such an ionic complexation is further evidenced by the proton nuclear magnetic resonance (^1^H NMR) spectral comparison among betaine, PAA, and PAA/betaine dissolved in D_2_O (Fig. 2b; see the whole spectra in Supplementary Fig. 11), in which the complexation-related H_1_, H_2_ resonance peaks from betaine and H_a_ peak from PAA all show remarkable chemical shifts. Furthermore, as presented in Fig. 2c, the pH of 0.1 M PAA (with respect to monomer concentration) in water is 2.18, while the pH of 0.1 M betaine is 6.5. Mixing PAA and betaine leads to pH values higher than that of PAA, suggesting that in PAA/betaine elastomer, betaine slightly deprotonates the carboxylic acid groups of PAA, and protons are the main charge carriers for ionic conductivity.

We further conducted iso-strain–stress relaxation experiments to study the relationship between stress dissipation and temperature^42^. As shown in Fig. 2d, a higher temperature is prone to promote the faster stress relaxation of PAA/betaine elastomer with a smaller residual stress. A stretched exponential function model was employed to study the stress relaxation kinetics with a theoretical prediction (Supplementary Table 1). ln(τ*) as a function of 1000/*T* was plotted and fitted and an apparent activation energy of 59.6 kJ mol^−1^ was calculated by the Arrhenius equation. Such a value is much smaller than a typical covalent bond dissociation energy (*E*_a_(C−C) ≈ 350 kJ mol^−1^), implying that the viscoelastic behavior of PAA/betaine elastomer is dominated by non-covalent interactions. The supramolecular nature of PAA/betaine elastomer is also supported by the tensile curves with different stretching rates (Supplementary Fig. 12). A higher stretching rate leads to a higher modulus and more pronounced strain-stiffening effect, due to the delayed and sluggish response of chain disassociation^16^.

Temperature-variable FTIR spectra of the as-prepared PAA/betaine elastomer from 20 to 70 °C were recorded to evaluate the strength and thermal sensitivities of the abovementioned interactions. As shown in Fig. 2e, with temperature increase, *v*(COO^−^) shifts to higher wavenumbers and *v*(C–N) shifts to lower wavenumbers, suggesting the conversion of hydrated COO^−^ and PAA-complexed C–N to self-associated [N(CH_3_)_3_]^+^:[COO^−^] ion pairs^34,40^. Meanwhile, *v*(COOH) from H-bonded PAA shifts to higher wavenumbers along with the intensity increase of free COOH, suggesting that temperature increase causes the disassociation of dimeric COOH H-bonds into free COOH. The temperature-dependent wavenumber shifts of the three main peaks present all gradual changes in the studied temperature range (Supplementary Fig. 13), indicating their mild thermal response. Conclusively, the increasing temperature would lead to the enhancement of ionic complexation and weakening of H-bonding, resulting in the more pronounced deprotonation degree of PAA and thus improved proton conductivity. From the energetical view, the PAA dimeric H-bonding could be enthalpy-driven with temperature-induced weakening effect, while the enhanced betaine ionic complexation at elevated temperatures implies an uncommon entropy-driven physical crosslinking interaction, which may be caused by the desolvation events that give rise to large increases in translational entropy of solvent molecules^43^.

2DCOS spectra were further generated from the temperature-variable FTIR spectra, which can discern the sequential thermal response of different species^44^. On the basis of Noda’s judging rule with the consideration of both signs in synchronous and asynchronous spectra (Fig. 2f), the responsive order of different groups to temperature increase is 1392 → 1728 → 1639 → 1402 → 1701 cm^−1^ (→ means prior to or earlier than; see determination details in Supplementary Table 2), that is, *v*(C–N) (betaine–betaine complex) → *v*(COOH) (free PAA) → *v*(COO^−^) (betaine) → *v*(C–N) (betaine–PAA complex) → *v*(C = O) (H-bonded PAA). This sequence suggests a thermal sensitivity order of betaine–betaine ionic complex > betaine–PAA complex > PAA dimeric H-bonds, which primely consolidates our hypothesis about interaction strength in Fig. 1c (weak interaction has a higher thermal sensitivity).

### Strain-stiffening mechanism discussion

**Fig. 3:**
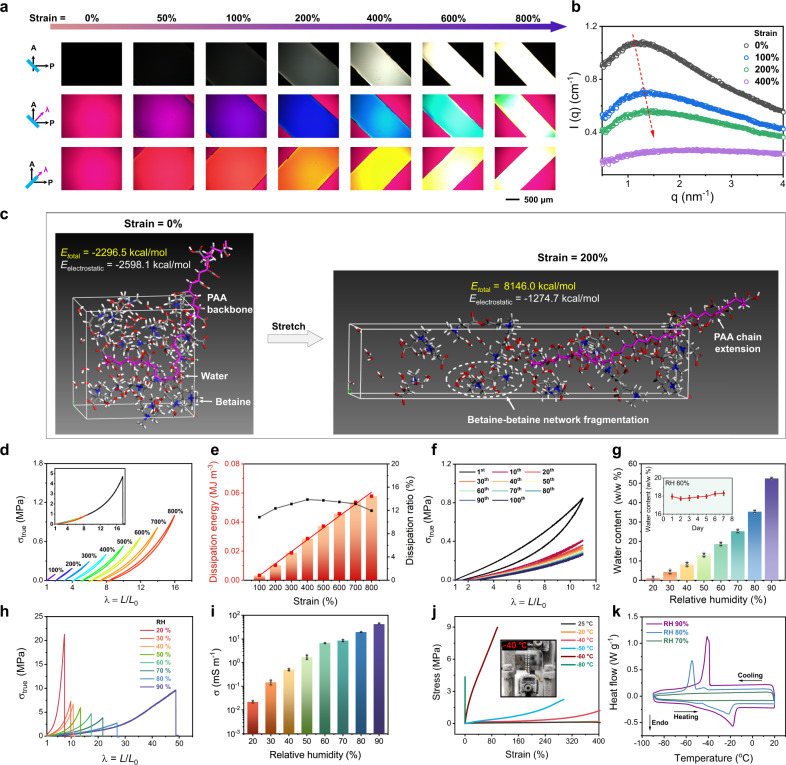
Strain-stiffening mechanism, elasticity, moisture-preserving, and anti-freezing properties of PAA/betaine elastomer. **a** Polarized optical images of PAA/betaine elastomer with increasing strains. The film thickness is 0.5 mm, and from top to bottom are the polarized modes in the absence of tint plate, and in the presence of 530 nm tint plate at azimuth angles of −45° and 45°, respectively. The observed orientation is parallel to the stretching direction. **b** SAXS scattering intensity (*I*) vs scattering vector (*q*) profiles of PAA/betaine elastomer with different strains. **c** Molecular dynamics simulation of PAA/betaine elastomer before and after stretching (PAA with 20 repeating units + 20 betaine + 50 H_2_O). With stretch, PAA chain extends, and the total energy significantly increases to a positive value indicating a metastable state. The increase of electrostatic energy is mainly due to betaine–betaine network fragmentation. **d** Successive tensile loading–unloading curves of PAA/betaine elastomer as stretched to different strains. The inset is the overlapped curves with single tensile curve to break. **e** Corresponding strain-dependent dissipation energy and dissipation ratios. **f** Cyclic tensile curves of PAA/betaine elastomer at a fixed maximum strain of 1000% for uninterrupted 100 cycles (tensile speed = 100 mm min^−1^). **g** Humidity-dependent water content changes of PAA/betaine elastomer at 25 °C. The inset is the water content changes when exposed to RH 60% for 7 days. **h**, **i** Mechanical properties and ionic conductivities of PAA/betaine elastomers equilibrated at different humidities. **j** DMA tensile curves of PAA/betaine elastomers at different temperatures. The inset picture shows that PAA/betaine elastomer remains elastic at −40 °C. **k** DSC heating and cooling curves of PAA/betaine elastomers equilibrated at RH 70, 80, and 90%. Data in (**g**, **i**) are presented as mean values ± SD, *n* = 3 independent elastomers.

To elucidate the strain-stiffening nature of PAA/betaine elastomer, we observed the strain-induced orientation changes with polarized optical microscopy. As shown in Fig. 3a, interference colors gradually appeared from 50% strain, and became stronger and stronger with increasing retardations after 100% strain. This observation accords with the initial slight decrease of differential modulus between 0 and 70% strain (Fig. 1e), in which supramolecular betaine network may be first destroyed and PAA network is stretched yet not stressed leading to the slight softening of the elastomer. After 100% strain, betaine network has been fully fragmented and the self-associated PAA chains with dimeric H-bonds are more and more extended resulting in the unidirectional chain alignment along the stretching direction and thus a stiffened material.

Small-angle X-ray scattering (SAXS) method was also employed to investigate the microstructural evolution of PAA/betaine elastomer. As shown in Fig. 3b, due to the thinner sample thickness with stretch, the scattering intensity of PAA/betaine elastomer decreases as the strain ratio increases. Interestingly, a broad peak between 1 and 2.5 nm^−1^ can be identified in all the samples, which is attributed to the ion aggregation of betaines^45^. With stretch, the peak position moves toward a larger *q* value, suggesting that the mean spacing between ion aggregates (*d*-spacing = 2*π*/*q*) becomes shorter, corresponding to the gradual approaching of fragmented betaine networks to each other forced by the aligned PAA chains. To further profile the strain-stiffening process, we made a molecular dynamics simulation of this process by stretching a periodic amorphous cell containing one 20-repeating-unit PAA chain, 20 betaine molecules, and 50 water molecules (same to the used AA:betaine:H_2_O molar ratio). As shown in Fig. 3c, at the undeformed state, PAA appears as a random-coil chain surrounded by the clustering betaine and water molecules, corresponding to an energetically stable soft network of PAA/betaine elastomer (total energy = −2296.5 kcal mol^−1^; electrostatic energy = −2598.1 kcal mol^−1^). As stretched to 200% strain, the PAA chain is strongly extended along the stretching direction, and betaine–betaine network is fragmented with electrostatic energy increasing to −1274.7 kcal mol^−1^. Note that the total energy also increases significantly to 8146.0 kcal mol^−1^, suggesting that the stretched state of the elastomer is metastable, which would autonomously recover to the initial state when released.

Overall, the above polarized optical observations as well as in situ SAXS and molecular dynamics simulation results together consolidate the hypothesis of dual-network design and the key role of supramolecular competing zwitterionic network in the observed strain-stiffening behavior of PAA/betaine elastomer. Moreover, such strain-stiffening phenomenon may also be understood with the classical rubber elasticity theory. For an ideal network with constant volume (*V*) and no energetic contribution to elasticity, the force *f* = −*T*(∂*S*/∂*L*)_*T*,*V*_, where *T* is the temperature, *S* the entropy, and *L* the length^46^. In other words, the generated force with stretch is mainly determined by the strain-dependent entropy loss of the whole system. In the as-prepared PAA/betaine elastomer, the curling, folding, wrapping, and collapsing of PAA chains as well as random clustering of betaines both contribute to a large conformational entropy^47^. Stretching would always decrease the entropy of PAA chains; however, the entropy-driven reorganizing nature of the betaine clustering network would compensate for the entropy loss of the whole system at small strains, while at large strains, fragmentation of betaine network occurs leading to more remarkable entropy loss. It is thus reasonable that a small applied force is enough to stretch the elastomer at small strains, while a larger force is required to stretch it more, corresponding to the apparent strain-stiffening behavior.

### Elasticity, moisture-preserving, and anti-freezing properties

In addition to high stretchability and strain-stiffening, PAA/betaine elastomer is also highly elastic with very rapid recovery. As presented in Fig. 3d, cyclically stretching the elastomer to increasing strains (100–800%) reproduced almost identical curves to the single tensile curve to break, and the elastic recovery ratio can reach 97.9 ± 1.1% as released from 100 to 800% strains. The hysteresis area as represented by the calculated dissipation energy increases almost linearly with strain, and corresponding dissipation ratios are rather small (<14%) (Fig. 3e). This phenomenon corresponds to the rapid elastic recovery of PAA/betaine elastomer with a low hysteresis, which may be related to the fast reformation of supramolecular betaine chain network in the unloading process^48^. This is understandable that betaine–betaine crosslinking in the elastomer is entropy-driven, so that the reformation of betaine chain network could autonomously occur with fast crosslink dynamics. To study its anti-fatigue behavior, the PAA/betaine elastomer was subjected to 100 consecutive loading–unloading cycles at a maximum strain of 1000% as shown in Fig. 3f. It is noted that after the first cycle (irreversible network rearrangement or physical bonding dissociation may occur in the first cycle), the tensile curves became narrower and almost unchanged until a steady state was reached in the following cycles, suggesting again the good elastic recovery of PAA/betaine elastomer even released from very large strains.

In nature, many plants accumulate zwitterionic osmolytes like betaine and proline to prevent water crystallization and adjust water stress to help them survive in subzero and water-deficit environments^49^. Similarly, in the case of PAA/betaine elastomer, the abundant presence of betaine brings excellent moisture-preserving and anti-freezing properties. As demonstrated in Fig. 3g and Supplementary Fig. 14, the as-prepared elastomer with the AA:betaine:H_2_O molar ratio of 1:1:2.5 has an almost equal water content of 21 wt% with the elastomer equilibrated at RH 60%; increasing environmental humidity would correspondingly increase the equilibrium water content and vice versa. Moreover, at the constant humidity of RH 60%, the water content of PAA/betaine elastomer is almost unchanged for a long time (7 days), suggesting the excellent moisturizing ability of betaine. As shown in Fig. 3h, the equilibrated PAA/betaine elastomer becomes much more stretchable (up to 4870% elongation at RH 90%) with a decreased tensile strength as the humidity increases. Such a trend was also observed by simply increasing the feed molar ratio of water in the as-prepared PAA–betaine samples (Supplementary Fig. 15). This is reasonable because a high amount of water as a plasticizer would weaken both the PAA dimeric bonds and betaine ionic complexes in PAA/betaine elastomer, which is supported by humidity-dependent ATR-FTIR and second derivative spectral analyses (Supplementary Fig. 16). Moreover, a higher humidity also induces a significant increase of proton conductivity from 0.02 mS m^−1^ at RH 20% to 42.2 mS m^−1^ at RH 90% (Fig. 3i). The freezing resistance of PAA/betaine elastomer is evidenced from the tensile stress–strain curves at different temperatures (Fig. 3j). It was observed that the PAA/betaine elastomer maintained prominent elasticity at a low temperature as −40 °C and became brittle lower than −50 °C. DSC detected an apparent freezing point of mobile water at −41.1 °C for the elastomer equilibrated at RH 90% and two freezing points at −54.8 and −44.1 °C for the sample equilibrated at RH 80% (Fig. 3k). No apparent freezing points were detected for the samples equilibrated at lower humidities than 70%, indicating that all the water molecules at these conditions are physically bound in the elastomer network.

### Self-healing, water-processable, and adhesive properties

**Fig. 4:**
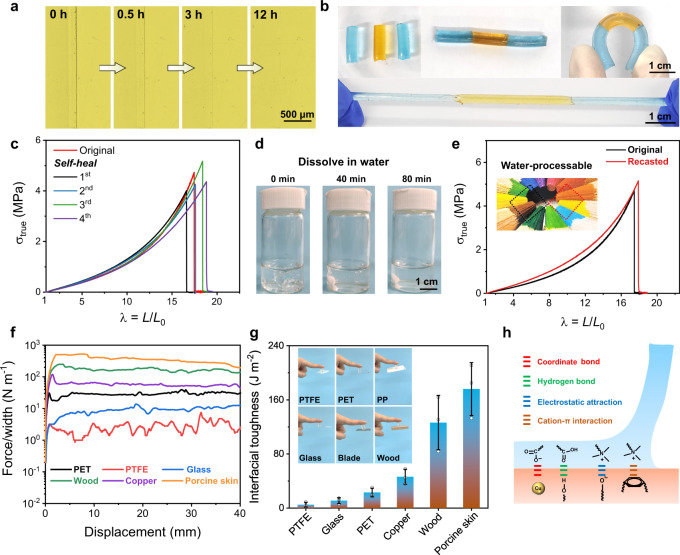
Self-healing, water-processable, and adhesive properties of PAA/betaine elastomer. **a** Self-healing process of the PAA/betaine elastomer was observed by the optical microscope. A scar autonomously healed after 12 h at RH 80%. The experiment was repeated three times independently with similar results. **b** Three individual PAA/betaine elastomer blocks healed together to withstand bending and stretching. The elastomers were colored blue and orange with methylene blue and semixylenol orange, respectively. **c** Tensile curves of the original elastomer and those after successive four self-healing cycles. **d** PAA/betaine elastomer can be dissolved in water in 80 min. **e** Tensile curves of the original and recasted PAA/betaine elastomer films at RH 60% indicating its water reprocessability. **f**, **g** Ninety-degree peeling curves and corresponding interfacial toughness of PAA/betaine elastomer with different substrates (peeling rate = 50 mm min^−1^). Data are presented as mean values ± SD, *n* = 3 independent elastomers. **h** Schematic adhesion mechanisms.

Owing to its supramolecular nature, PAA/betaine is also self-healable as immersed in a high-humidity environment. As shown in Fig. 4a, the scar on PAA/betaine film completely disappeared after 12 h at room temperature and RH 80%, assisted by the mobile water to promote the regeneration of all physical bonds. Similarly, several cut PAA/betaine blocks also healed together at this condition to withstand bending and stretching (Fig. 4b and Supplementary Movie 2). Figure 4c shows the tensile curves of the original and healed samples for four times, which almost coincide indicating ~100% healing efficiency. Furthermore, PAA/betaine elastomer can also be easily recycled by dissolving in water and recasting in air. Since the elastomer is composed of a linear polymer and abundant small molecules, as displayed in Fig. 4d, a film can be rapidly dissolved in water in 80 min. Recasting the solution to evaporate water in the air at RH 60% reproduced all the original mechanical properties (Fig. 4e). Furthermore, PAA/betaine elastomer can also be fully dissolved in biologically relevant media (e.g., normal saline, phosphate-buffered saline (PBS) buffer, Dulbecco’s modified Eagle’s medium (DMEM) and RPMI-1640 culture media), and the recasted films remain transparent and highly stretchable (Supplementary Fig. 17).

In addition, as previously presented in Fig. 1e, the PAA/betaine elastomer is not only soft but also self-adhesive. Here, we evaluate its adhesion energy with various substrates via 90° peeling tests. As shown in Fig. 4f, g, the interfacial toughness between PAA/betaine elastomer and adherends (polytetrafluoroethylene (PTFE), glass, PET, copper, wood, and porcine skin) ranges from 5 J m^−2^ for PTFE to a maximum of 176 J m^−2^ for porcine skin. We attribute the strong adhesion of PAA/betaine elastomer on polar substrates to the presence of multiple interactions including metal coordination, hydrogen bonding, electrostatic attraction, and cation–π interactions, as illustrated in Fig. 4h^11^. The strongest adhesion occurs on porcine skin, which may be caused by both the rough surface of the skin and the wet interface that facilitates the penetration of PAA and betaine molecules leading to more physical interlocks.

### Multiple sensory capabilities of ionic skin

**Fig. 5:**
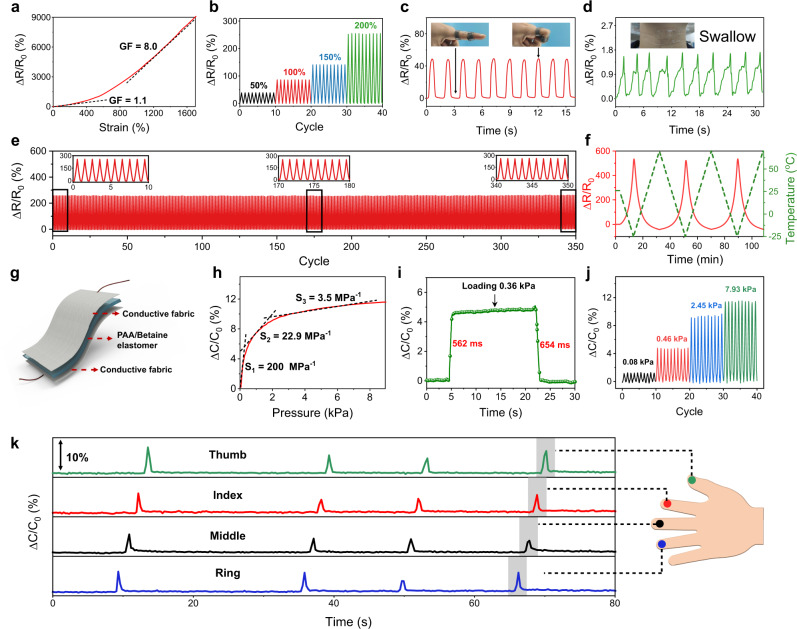
Sensing applications of PAA/betaine elastomer-based iontronic sensors. **a** Strain-dependent resistance changes of PAA/betaine elastomer. **b** Real-time response curves measured at fixed strains of 50, 100, 150 and 200% for ten cycles each. **c**, **d** The elastomer-based strain sensor senses human finger bending and swallowing movements. **e** Relative resistance variations of the elastomer-based strain sensor upon stretching to 200% strain for 350 cycles. **f** Temperature-induced real-time resistance changes of PAA/betaine elastomer between −25 and 70 °C. **g** Schematic structure for the PAA/betaine elastomer-based iontronic pressure sensor. **h** Pressure-dependent capacitance variations (loading speed: 5 mm min^−1^). S_1_, S_2_, and S_3_ are the calculated sensitivities. **i** Response time at the loading pressure of 0.36 kPa. **j** Dynamic loading/unloading curves at different pressures. **k** Synchronized capacitive response of four iontronic sensors adhered on a glove by pressing with different fingers.

As a unique mechanoresponsive and adhesive ionic skin, PAA/betaine elastomer is able to serve as a sensor to perceive a variety of external stimuli via electrical signals. For instance, as the elastomer is stretched, the resistance increases nonlinearly upward with strain (Fig. 5a). The gauge factors in the small and large strain ranges were calculated to be 1.1 and 8.0, respectively. Cyclically stretching PAA/betaine film to the fixed strains of 50, 100, 150, and 200% produced repeatable and strain-dependent resistance changes (Fig. 5b). The ability of PAA/betaine elastomers as strain sensors was demonstrated by directly adhering to the adhesive film on the human fingers and throat, and bending or swallowing produced reliable electrical response (Fig. 5c, d and Supplementary Fig. 18). In addition, the change in resistance of the elastomer exhibited excellent stability and repeatability during continuous stretching for 350 cycles at a fixed strain of 200% (Fig. 5e), indicating its prominent durability. Moreover, the proton conductivity of PAA/betaine elastomer is also sensitive to temperature, and the sensitivity can reach a very high value of 1.58% °C^−1^, higher than conventional polyacrylamide hydrogel-based ionic skin and a few commonly used salt solutions and ionic liquids (Supplementary Fig. 19). As shown in Fig. 5f, heating and cooling the elastomer film produced a very rapid response of resistance changes in a wide temperature range between −25 and 70 °C.

Furthermore, the PAA/betaine elastomer could also act as an iontronic capacitive sensor to detect pressure changes. As illustrated in Fig. 5g, the pressure sensor has a sandwich-like structure consisting of two elastic conductive fabrics as electrodes and PAA/betaine elastomer as the iontronic layer. When a normal force is applied to the sensor, the electrical double layer capacitance at the elastic electrolytic–electronic interface will correspondingly change^50^. We evaluated pressure sensitivity, response time, and repeatability to characterize the performance of PAA/betaine elastomer-based iontronic sensor. The sensitivity is defined as *S* = δ(∆*C*/*C*_0_)/δ*P*, which is the slope of the measured capacitance-pressure curve (Fig. 5h). As shown, the fabricated sensor could operate over a wide pressure range with a sensitivity of 200 MPa^−1^ in the low-pressure range (<0.5 kPa), and 22.9 and 3.5 MPa^−1^ in the high-pressure range, typical for iontronic sensors^50^. Upon loading a pressure of 0.36 kPa, the response time appeared to be very short within 700 ms (Fig. 5i). As shown in Fig. 5j, dynamically increasing the applied pressure also generated reliable and repeatable capacitance changes. To further characterize its durability, the iontronic sensor was subjected to more than 450 cycles of loading/unloading experiments under the same pressure; as shown in Supplementary Fig. 20, the highly reproduced capacitance signals strongly confirm the robust performance of the sensor. As a demonstration, we adhered four PAA/betaine elastomer-based iontronic sensors on a glove, and pressing an object with the ring, middle, index, and thumb fingers in sequence presented real-time and sequential response from the synchronized four data-collecting channels (Fig. 5k). All the above results suggest the great potential of PAA/betaine elastomer-based ionic skin in detecting timely strain, temperature, and pressure changes that are important in wearable electronics and smart fabrics.

## Discussion

In this paper, we report, to the best of our knowledge, the first example of ionic skins to mimic the all-around sensory, self-healing, and strain-stiffening properties of natural skin. The unique mechanoresponsive ionic skin was readily achieved via a dual-network design by introducing an entropy-driven supramolecular zwitterionic competing network to the H-bonded polycarboxylic acid network to mimic the roles of soft elastin network and stiff collagen fibers in natural skins, respectively. Compared to previously reported ionic skin materials involving hydrogels, organohydrogels, ionogels, and ionic conductive elastomers, the present elastomer shows extraordinary mechanical and optical properties in terms of strain-stiffening, full self-healing, ultrahigh stretchability, excellent elastic recovery, high transparency, air stability (for gels, the ability of solvent retention in the air), frost resistance, and self-adhesion (see a rough comparison with several typical ionic skin materials in Table 1; extended comparison can be found in Supplementary Table 3). Intriguingly, the combination of two competing networks attributed from three dynamic interactions with different interacting strengths defeats the inherent conflict among elasticity, self-healability, and strain-stiffening for stretchable ionic conductors. This work not only lays the mechanistic foundation for interpreting the dual crosslinking network-related nonlinear mechanoresponsive behavior but also paves the way for designing robust materials with skin-like sophisticated sensory and mechanical properties. We anticipate our strategy may inspire a series of biomimetic materials with a variety of applications in sensors, wearable electronics, smart textiles, human–machine interfacing, and so on.

**Table 1:** A rough comparison of the overall performance between this work and previously reported typical ionic skin materials.

Ionic skin materials	Mechanoresponse	Self-healing	Max. strain (%)	Elastic recovery ratio (%)	Transparency (%)	Air stability	Anti-freezing (°C)	Adhesion	Refs.
PAAm/salt hydrogel	Softening	None	~800	~100	>95	Poor	−17	None	^6,48,52^
ACC/PAA/alginate hydrogel	Softening	20 min, 100%	>1000	Poor	Opaque	Poor	None	None	^18^
PVA/MXene hydrogel	Softening	Fast, 100%	>3400	Poor	Opaque	Poor	None	None	^53^
PDMAPS/clay hydrogel	Softening	24 h, 98%	1500	NA	98.8	Poor	NA	Yes	^11^
P(SPMA-*r*-MMA) organohydrogel	Softening	3 h, 98.3%	2636	~89	~100	Good	NA	NA	^54^
PMMA-*r*-BA/ionogel	Softening	NA	850	96.1	98.5	Good	NA	NA	^13^
PDMAPS ionogel	Softening	48 h, 100%	>10,000	63	>90	Good	<−10	NA	^14^
PIL-based ionogel	Stiffening	None	1390	97	~95	Good	−75	NA	^55^
P(MEA-*co*-IBA) elastomer	Stiffening	24 h, 30–40%	1744	95	90	Good	−14.4	Yes	^56^
PAA/betaine elastomer	Stiffening	12 h, 100%	1600	97.9	99.7	Good	−40	Yes	This work

## Methods

### Materials

Betaine, dimethylglycine, l-proline, sarcosine, TMAO, and methylene blue were obtained from Shanghai Titan Technology. AA and semixylenol orange were purchased from Aladdin Chemical. Irgacure 2959 was purchased from TCI chemical. PAA (average *M*_W_ ≈ 100,000 g mol^−1^, 35 wt% in H_2_O), acrylamide, *N,N*′-methylene bisacrylamide, ammonium persulfate, *N,N,N*′*,N*′-tetramethylethylenediamine, and LiBr were purchased from Sigma-Aldrich. NaCl and CH_3_COOH were purchased from Sinopharm Chemical Reagent. RPMI-1640 and DMEM (high glucose) culture media were purchased from Genom Co. Ltd. PBS and normal saline solutions were freshly prepared in the laboratory.

### Preparation of PAA/zwitterion elastomers

AA was purified by passing through a basic alumina column to remove inhibitors. The reaction precursor was prepared by mixing AA, zwitterion, H_2_O (molar ratio, AA:zwitterion:H_2_O = 1:1:2.5) with Irgacure 2959 as photoinitiator (1:500 molar ratio to the monomer). The precursor was then poured into a sealed glass mold with silicone spacer, and polymerized by 360 nm UV irradiation for 30 min.

### Preparation of PAA/betaine elastomer-based iontropic pressure sensor

PAA/betaine elastomer-based iontropic sensor was fabricated by sandwiching a dielectric layer of PAA/betaine elastomer (thickness: 2 mm) between two elastic conductive fabrics (LessEMF; surface resistivity <1 Ω sq^−1^; thickness: 0.40 mm). Two electrodes were connected to the conductive fabrics to monitor the pressure-induced real-time capacitance changes with a multichannel source meter (DAQ6510, Keithley).

### Characterizations

The size distributions of AA/betaine/H_2_O mixture, AA/H_2_O mixture, and saturated betaine solution were determined by DLS (Malvern Zetasizer Nano ZS) at a backscatter angle of 173°. Tensile tests of the elastomers were carried out on a universal testing machine (UTM2103, Shenzhen Suns technology) at a rate of 100 mm min^−1^ unless otherwise stated. All the measured samples have a length of 8 mm, a thickness of 0.5 mm, and a width of 5 mm. The transparency of the film (thickness ~100 μm) was evaluated with a UV–Visible spectrophotometer (Lambda 950, PerkinElmer). ATR-FTIR spectra were collected on a Nicolet iS50 spectrometer with the ATR diamond crystal. Thermogravimetry analysis (TGA) was performed on TA TGA550 via scanning a temperature range from 30 to 300 °C under airflow (heating rate = 10 °C min^−1^). Differential scanning calorimetry (DSC) heating and cooling curves were collected using TA DSC250 scanning from −90 °C to 20 °C at a scanning rate of 5 °C  min^−1^ under nitrogen flow. The stress relaxation measurements with the strain of 100% and tensile tests at specific temperatures were performed on a TA Q800 dynamic mechanical analyzer (DMA). Ninety-degree peeling tests were carried out using the 90° peel test apparatus equipped on a vertical dynamometer (ESM303, MARK-10). Before the test, a rectangular copper foil of 4 × 1 cm^2^ was laminated on the elastomer/substrate and preloaded by 700 g weight for 30 min. The PAA/betaine elastomer film was then delaminated perpendicularly to the substrate at a rate of 50 mm min^−1^. Resistance changes of PAA/betaine elastomer-based sensors were monitored by a multimeter (Tektronix, DMM 4050).

### Simulation of stress relaxation results

Kohlrausch–Williams–Watts function was employed to simulate the stress relaxation behavior

1 $$\frac{\sigma }{{\sigma }_{0}}=\exp \left(-{\left(\frac{t}{\tau }\right)}^{\beta }\right)$$

where τ* is the characteristic relaxation time at which *σ*/*σ*_0_ is the numerical value of 1/*e*. The exponent *β* (0 < *β* < 1) is the breadth of the stress relaxation time distribution. τ* and *β* are the fitting parameters, as shown in Supplementary Table 1.

### Temperature-variable FTIR measurement

The sample was prepared as follows: ~200 µL of PAA/betaine precursor was cast on a CaF_2_ tablet and then covered with another tablet. The tablets were then irradiated under UV light (365 nm) for 1 h at room temperature to obtain PAA/betaine elastomer film with suitable thickness for transmission IR measurement. The sample was further put in a chamber at room temperature and RH 60% for 24 h to reach equilibrium. For temperature-controlled measurement, the sealed sample was heated from 20 to 70 °C with an interval of 5 °C in the transmission mode on a Nicolet iS50 FTIR spectrometer.

### Two-dimensional correlation spectroscopy

The temperature-dependent FTIR spectra of PAA/betaine elastomer from 20 to 70 °C were used for performing 2D correlation analysis. 2D correlation analysis was carried out using the software 2D Shige ver. 1.3 (©Shigeaki Morita, Kwansei-Gakuin University, Japan, 2004–2005), and was further plotted into the contour maps by Origin program, ver. 9.8. In the contour maps, warm colors (red and yellow) are defined as positive intensities, while cold colors (blue) as negative ones.

### Small-angle X-ray scattering

SAXS experiments were performed at the SSRF beamline BL16B (Shanghai, China) at an X-ray energy of 10.0 keV with a wavelength of *λ* = 1.24 Å. Samples were measured perpendicular to the beam with the sample-detector distance of 1.87 m to cover the scattering vector *q* range from 0.1 to 6 nm^−1^ (*q* is the scattering vector, *q* = (4*π*/*λ*)sin(*θ*); 2*θ* is the scattering angle). The scattering patterns were obtained with a short exposure time (120 s), and air as the background was subtracted. The SAXS patterns were radially averaged to obtain the intensity profiles.

### Molecular dynamics simulation

A periodic model of PAA/betaine elastomer containing one PAA chain (20 repeating units), 20 betaine molecules, and 50 water molecules was constructed in the Amorphous Cell module of Materials Studio, ver. 2019. The structure optimization and the calculation of potential energies were performed in the Forcite module. Cell parameter adjustment and re-optimization were employed to simulate the stretching process.

### Cytotoxicity tests of PAA/betaine elastomer

The cytotoxicity of PAA/betaine elastomer was evaluated with HeLa cells (cervical cancer) and HepG2 (human hepatocellular carcinomas) cells using MTT (3-(4,5-dimethylthiazol-2-yl)-2,5-diphenyltetrazolium bromide) assays. The cells were seeded in 96-well plates at a density of 1 × 10^4^ cells/well, and then cultured in 5% CO_2_ at 37 °C for 24 h. The original medium was replaced with a fresh culture medium containing PAA/betaine elastomer at a final concentration of 0–2 mg/mL and incubated for 12 and 24 h, respectively. MTT (10 μL, 0.5 mg/mL) was added to each well of the 96-well assay plate for 4 h at 37 °C. After DMSO (100 μL/well) was added, the absorbance was measured at 450 nm using a microplate reader.

### Reporting summary

Further information on research design is available in the Nature Research Reporting Summary linked to this article.

## Supplementary information

Supplementary Information

Description of Additional Supplementary Files

Supplementary Movie 1

Supplementary Movie 2

Reporting Summary

## Data Availability

The data that support the findings of this study are available within the article and its Supplementary information files.
