# Supplementary material for: Skin-like mechanoresponsive self-healing ionic elastomer from supramolecular zwitterionic network
Source: Nat Commun. 2021 Jul 2;12:4082. doi: 10.1038/s41467-021-24382-4 (PMC8253733; doi:10.1038/s41467-021-24382-4)
Supplement: Supplementary file 1 — Supplementary Information [file 41467_2021_24382_MOESM1_ESM.pdf]

Supplementary Information for

Skin-like mechanoresponsive self-healing ionic elastomer from supramolecular zwitterionic network

Wei Zhang[1], Baohu Wu[2], Shengtong Sun[1,*], and Peiyi Wu[1,*]

[1]State Key Laboratory for Modification of Chemical Fibers and Polymer Materials, College of Chemistry, Chemical Engineering and Biotechnology & Center for Advanced Low-dimension Materials, Donghua University, 2999 North Renmin Road, Shanghai 201620, China

*Correspondence to: shengtongsun@dhu.edu.cn; wupeiyi@dhu.edu.cn

[2]Jülich Centre for Neutron Science (JCNS) at Heinz Maier-Leibnitz Zentrum (MLZ) Forschungszentrum Jülich, Lichtenbergstr. 1, 85748 Garching, Germany

This material includes

Extended Methods

Suppl. Fig. 1 Photos of PAA/zwitterion proton-conductive elastomers

Suppl. Fig. 2 UV-Vis transmittance spectrum of PAA/betaine elastomer film

Suppl. Fig. 3 DLS size distribution of saturated betaine solution

Suppl. Fig. 4 Photos of betaine/H_2O, AA/betaine, AA/H_2O, and AA/betaine/H_2O (1:1:2.5) mixtures

Suppl. Fig. 5 Tensile curves of five PAA/zwitterion elastomers prepared in three batches

Suppl. Fig. 6 Initial moduli of PAA/zwitterion elastomers

Suppl. Fig. 7 Effect of zwitterions on the enthalpic dimeric H-bonds of PAA

Suppl. Fig. 8 Tensile curves of PAA/betaine elastomers with different molar ratios of AA and betaine

Suppl. Fig. 9 Tensile curves of PAA/LiBr and PAA/CH_3COOH elastomers

Suppl. Fig. 10 Cytotoxicity tests of PAA/betaine elastomer

Suppl. Fig. 11 ^1H NMR spectra of PAA, betaine and their mixture dissolved in D_2O

Suppl. Fig. 12 Tensile curves of PAA/betaine elastomer at different stretching rates

Suppl. Fig. 13 Temperature-dependent wavenumber shifts of ν(C-N), ν(COO^-), and ν(COOH)

Suppl. Fig. 14 TGA curves of the as-prepared PAA/betaine elastomer and the elastomer equilibrated at RH 60%
Suppl. Fig. 15 Tensile curves of the as-prepared PAA/betaine elastomers with different water contents
Suppl. Fig. 16 ATR-FTIR spectra of PAA/betaine elastomer equilibrated at different humidities
Suppl. Fig. 17 Recyclability of PAA/betaine elastomer in biologically relevant media
Suppl. Fig. 18 Resistance variations of PAA-betaine elastomer adhered on human finger at different bending angles
Suppl. Fig. 19 Temperature-dependent resistance variations and corresponding gauge factors of ionic liquids, saline solutions, PAAm/NaCl hydrogel and PAA/betaine elastomer
Suppl. Fig. 20 Capacitance response curve of PAA/betaine elastomer under the dynamically applied pressure of 2.45 kPa for 450 cycles
Suppl. Table 1. Fitting parameters (τ^* and β) in the simulation of iso-strain–stress relaxation results
Suppl. Table 2. Final results of the multiplication of the signs of each cross-peak in 2DCOS synchronous and asynchronous spectra of PAA/betaine elastomer
Suppl. Table 3. Extended comparison of the overall performance between this work and previously reported polyzwitterion-based hydrogels and ionic skin materials
References S1-S22
Suppl. Movie 1. High elasticity, mechanical compliance, and adhesion of PAA/betaine elastomer
Suppl. Movie 2. Self-healing of PAA/betaine elastomer

Extended Methods

Preparation of PAAm/NaCl hydrogel. PAAm/NaCl hydrogel was prepared according to literature.[S1] Briefly, the gel precursor was prepared by dissolving AAm and NaCl into deionized water with molar concentrations of 2.2 M and 2.74 M, respectively. MBAA (0.06 wt%) and APS (0.17 wt%) with respect to the weight of AAm monomer were then added as the cross-linker and initiator, respectively. After degassing in a vacuum chamber, TEMED (0.25 wt%, with respect to the weight of AAm monomer) was added as an accelerator. The solution was poured into a glass mold, and cured by UV light for 20 min. The obtained hydrogel was further immersed in aqueous solution with the same concentration of NaCl for more than 24 h.

Two-dimensional correlation spectroscopy (2DCOS). 2DCOS, as a mathematical method, is very suitable for the investigation of the variations of chemical groups. Its basic principle was first proposed by Noda[S2, S3] and have been applied more and more widely to follow spectral variations under various external perturbations, such as time, temperature, pressure, concentration and other physical variables. By spreading the original spectral information along a second dimension, spectral resolution enhancement can be achieved, allowing the additional important information about molecular motions or conformational changes not readily visible in conventional analysis to be extracted.

2DCOS includes two types of correlation maps, synchronous spectrum which reflects simultaneous changes between two wavenumbers, and asynchronous spectrum which can significantly enhance the spectral resolution. The auto-peaks only appear along the diagonal in synchronous spectrum, while the cross-peaks can appear in both synchronous and asynchronous spectra. The judging rule of the sequence can be summarized as Noda's rule- that is, if the multiplication of the signs of cross-peaks (v_1, v_2, and assume $v_1 > v_2$) in synchronous and asynchronous spectra is positive, the change at v_1 may occur prior to v_2, and vice versa[S4,S5]. In Supplementary Table 2, "+" means the same signs in synchronous and asynchronous spectra, while "-" means different signs in synchronous and asynchronous spectra.

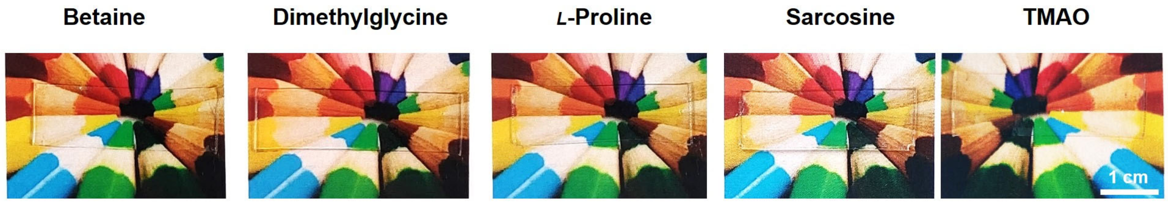

Supplementary Fig. 1. Photos of PAA/zwitterion proton-conductive elastomers indicating their good transparency.

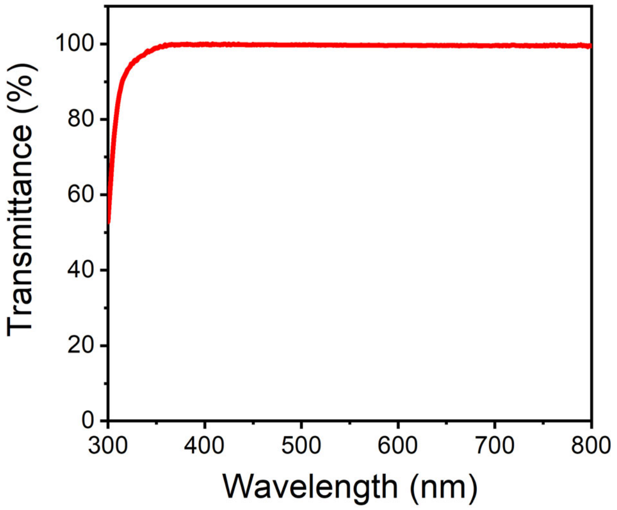

Supplementary Fig. 2. UV-Vis transmittance spectrum of PAA/betaine elastomer film (thickness ≈ 100 μm).

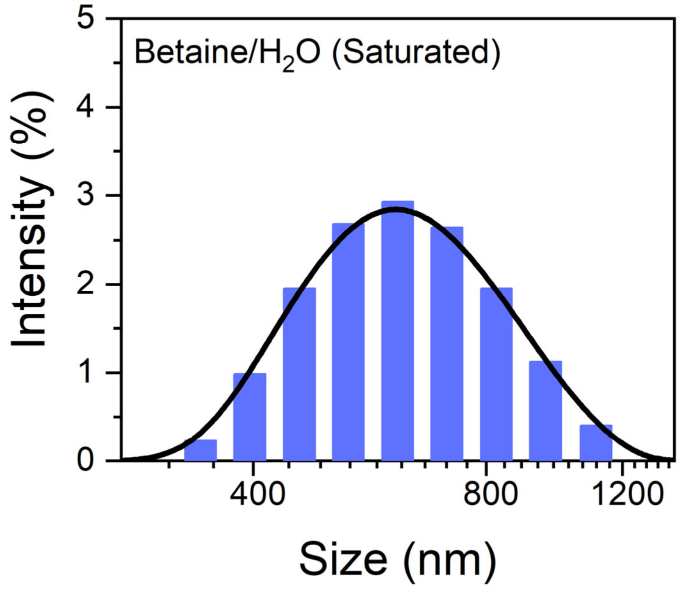

Supplementary Fig. 3. DLS size distribution of saturated betaine solution (~13.6 M). The peak at 615 nm comes from large betaine clustering aggregates.

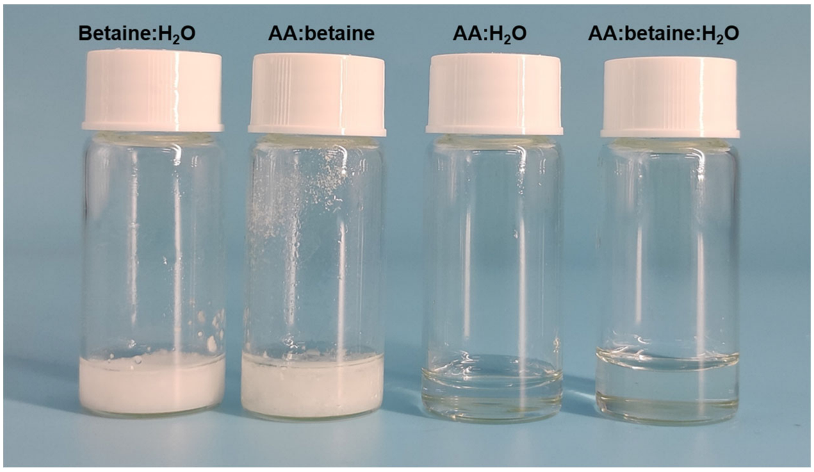

Supplementary Fig. 4. Photos of betaine/H_2O (1:2.5), AA/betaine (1:1), AA/H_2O (1: 2.5) and AA/betaine/H_2O (1:1:2.5) mixtures. Due to the interactions among AA, betaine and water, the enhanced cosolvency of the three components is observed.

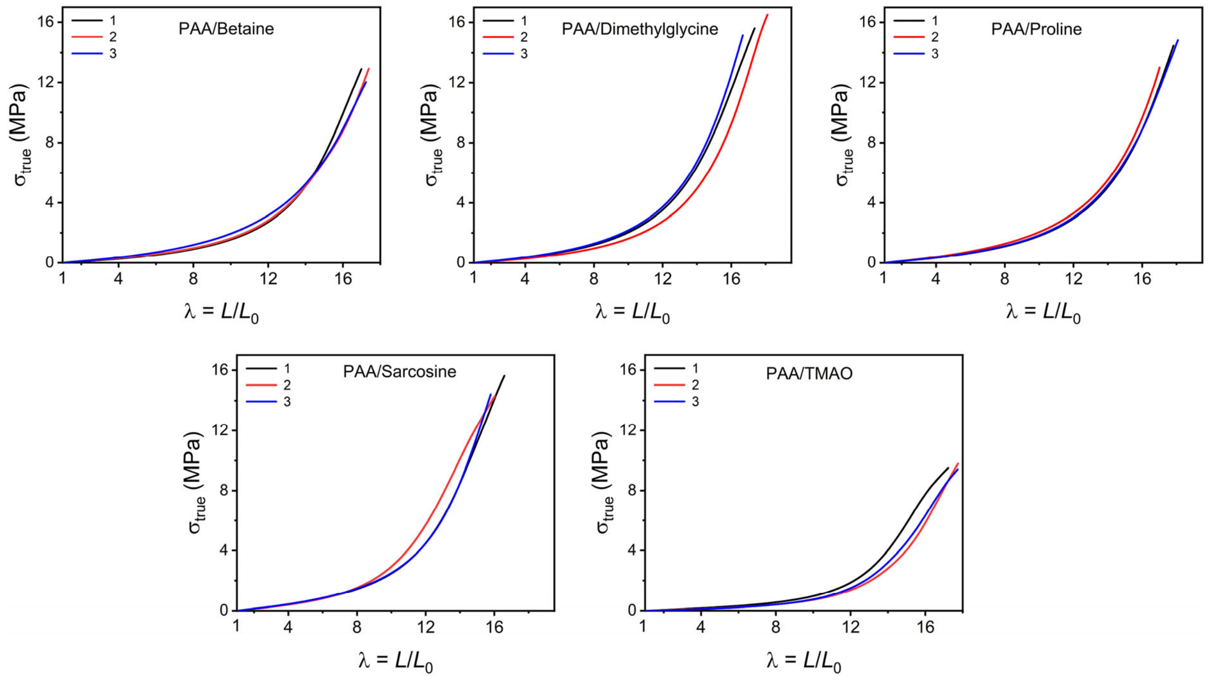

Supplementary Fig. 5. Tensile curves of the five PAA/zwitterion elastomers prepared in three batches suggest the good reproducibility of strain-stiffening response.

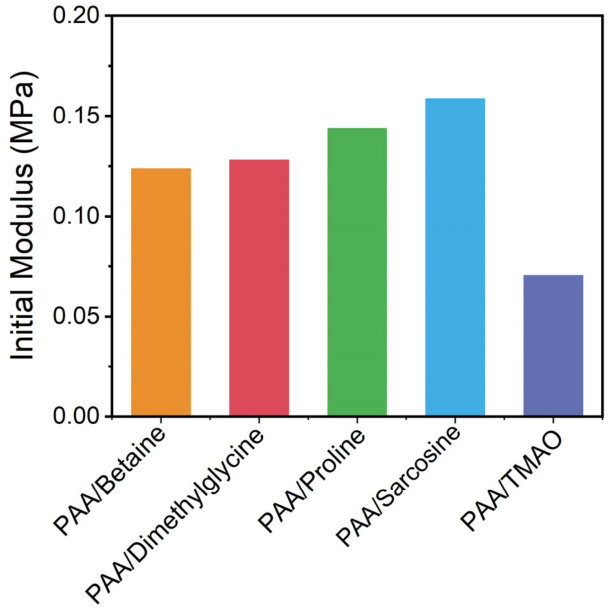

Supplementary Fig. 6. Initial moduli of PAA/zwitterion elastomers.

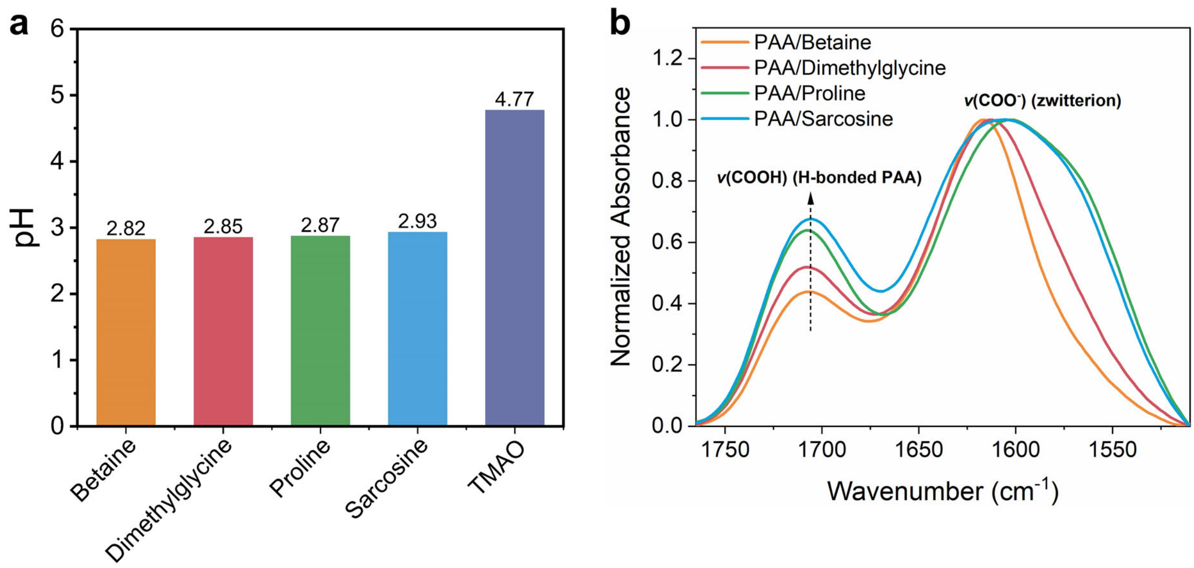

Supplementary Fig. 7. Effect of zwitterions on the enthalpic dimeric H-bonds of PAA. **a** pH values of 0.1 M PAA/zwitterion solutions by mixing PAA (with respect to monomer concentration), zwitterion, and water in the same molar ratio of 1:1:2.5. **b** Normalized ATR-FTIR spectra of PAA/zwitterion elastomers equilibrated at RH 60%.

In the cases of betaine, dimethylglycine, proline, and sarcosine, the pH values of PAA/zwitterion solutions are very close to each other, suggesting almost the same deprotonation degree of PAA. However, the relative amount of *v*(COOH) (H-bonded PAA) as normalized by the spectral intensity of *v*(COO^-) (zwitterion) increases in the order of betaine, dimethylglycine, proline, and sarcosine, corresponding to the formation of more H-bonded crosslinks. This order is in full accordance with the increment of the initial moduli of PAA/zwitterion elastomers (Supplementary Fig. 6), which may result from the reduced zwitterion-PAA interactions in the case of tertiary and secondary amines with a lower electropositivity. The lowest initial modulus of PAA/TMAO elastomer should be caused by the highest deprotonation degree of PAA (highest pH) resulting in much less H-bonded crosslinks than the other four zwitterions.

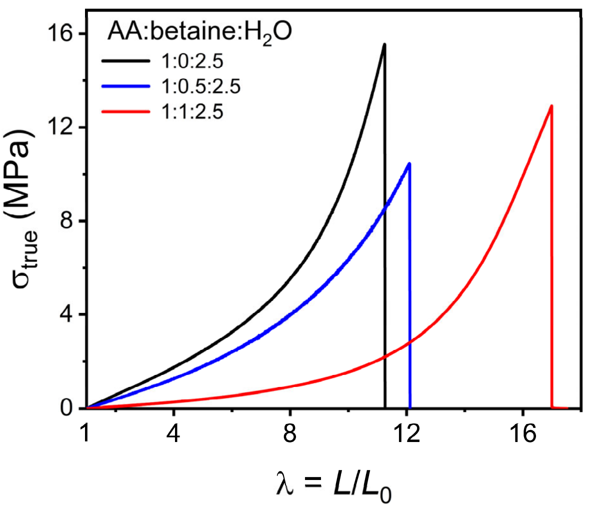

Supplementary Fig. 8. Tensile curves of PAA/betaine elastomers with different molar ratios of AA and betaine. Decreasing the ratio of betaine leads to more rigid elastomers, and the strain-stiffening effect and stretchability are also largely weakened.

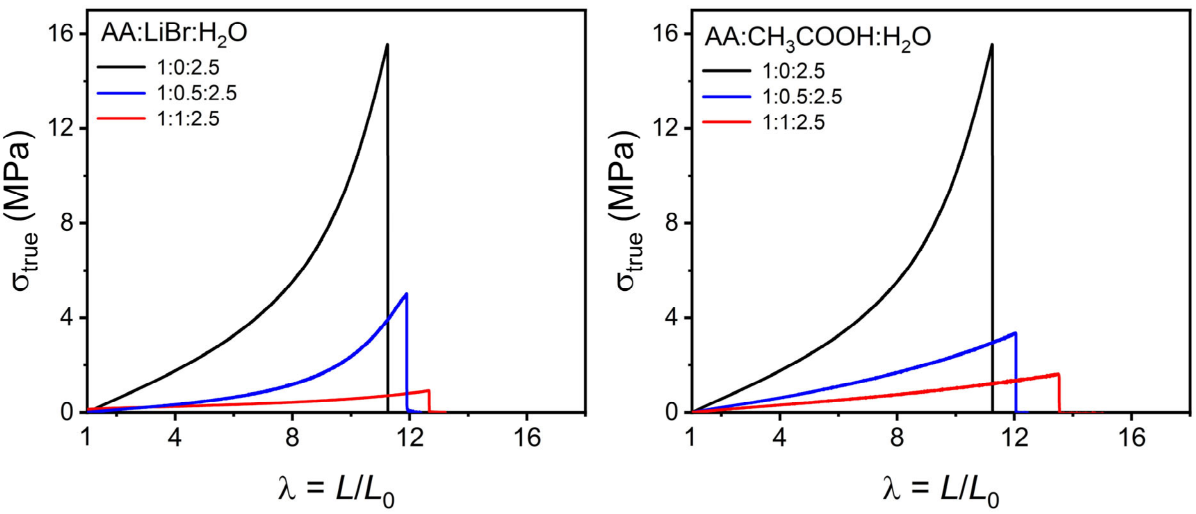

Supplementary Fig. 9. Tensile curves of PAA/LiBr and PAA/CH_3COOH elastomers with different molar ratios of AA and salt/acid. The PAA/LiBr and PAA/CH_3COOH elastomers show much weakened tensile strength and stretchability compared to PAA/betaine elastomer in the same molar ratio, and the strain-stiffening effect is also largely diminished. These two sets of control samples support the role of supramolecular betaine network in the strain-stiffening behavior.

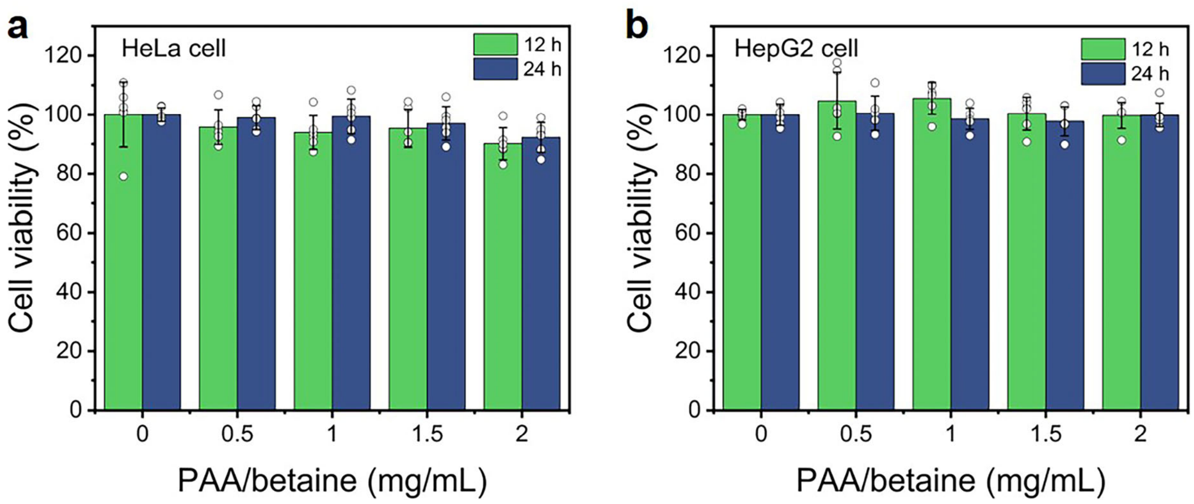

Supplementary Fig. 10. Cytotoxicity tests of PAA/betaine elastomer. Cell viability of **a**) HeLa and **b**) HepG2 cells incubated with different amounts of PAA/betaine elastomer for 12 h and 24 h, respectively. The PAA/betaine elastomer is proved to have very good biocompatibility. Data are presented as mean values ± SD, n = 6 independent solutions.

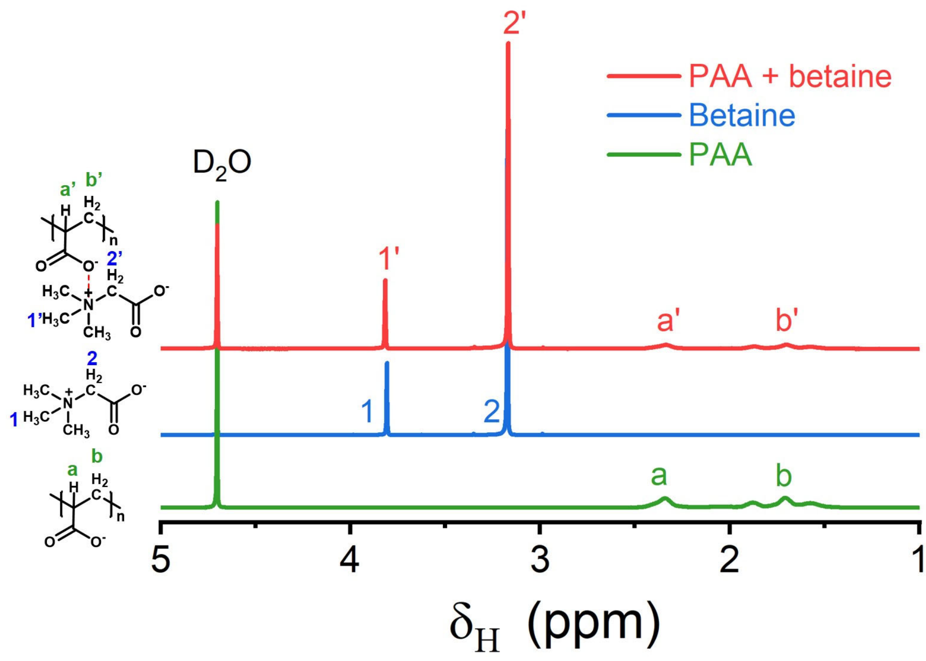

Supplementary Fig. 11. ^{1}H NMR spectra of PAA (M_w = 100,000 g mol^{-1}), betaine and their mixture dissolved in D_2O. The total solute concentrations are all 0.1 M.

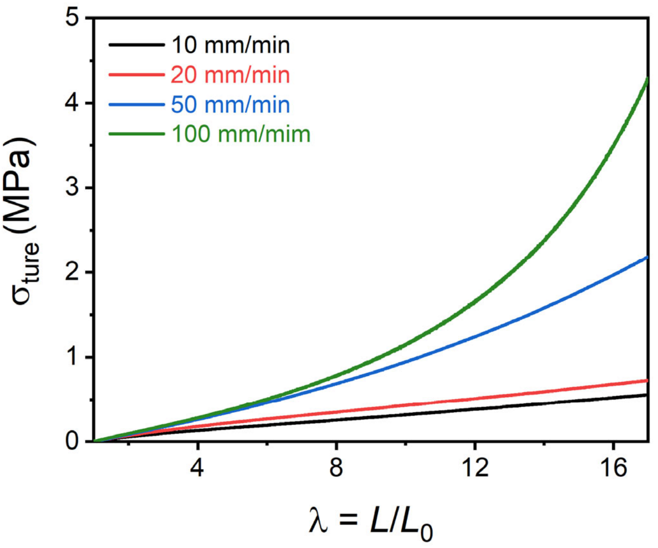

Supplementary Fig. 12. Tensile true stress-elongation curves of PAA/betaine elastomer at different stretching rates. A higher stretching rate leads to a high modulus and more pronounced strain-stiffening effect, proving the supramolecular nature of PAA/betaine elastomer. Notably, both PAA chain relaxation and betaine chain disassociation are strongly rate-dependent. A slowing stretching rate will give enough time for betaine chain fragmentation/reorganization and PAA chain rearrangement.

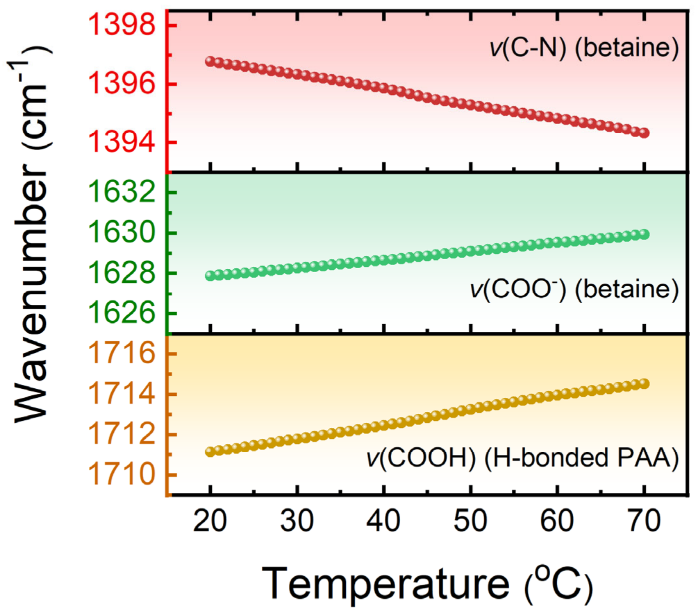

Supplementary Fig. 13. Temperature-dependent wavenumber shifts of *v*(C-N) (betaine), *v*(COO^-) (betaine) and *v*(COOH) (H-bonded PAA).

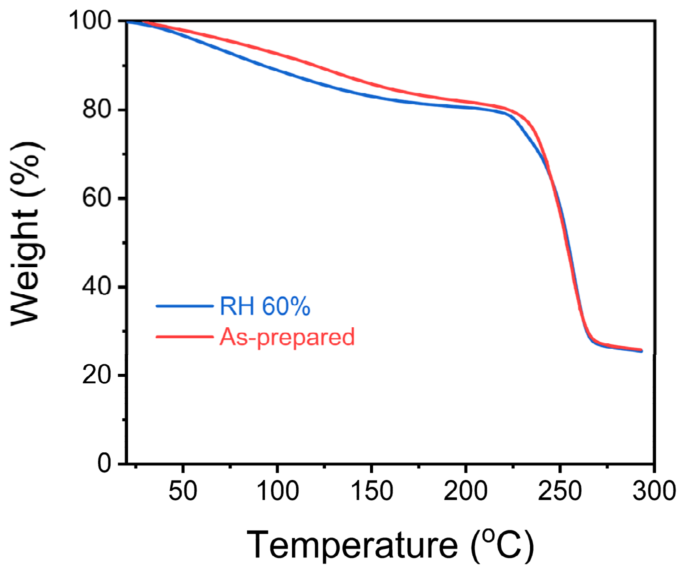

Supplementary Fig. 14. TGA curves of the as-prepared PAA/betaine elastomer and the elastomer equilibrated at RH 60% which show the almost equal water content of 21 wt%.

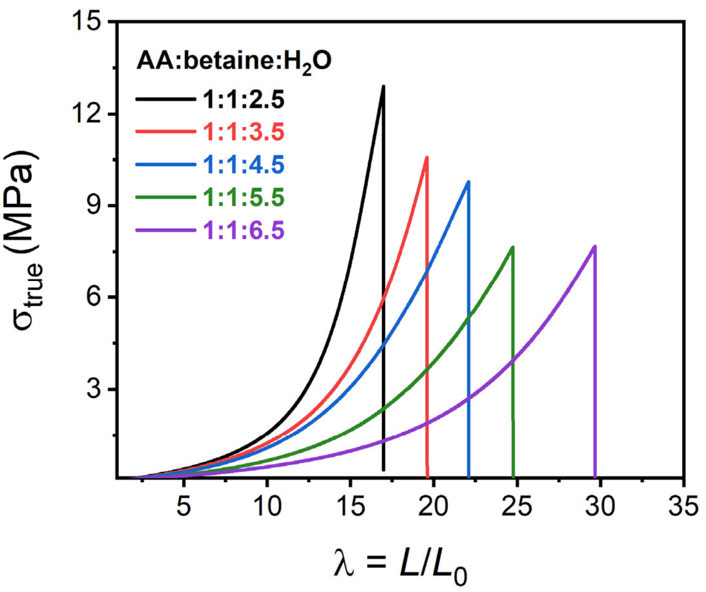

Supplementary Fig. 15. Tensile true stress-elongation curves of the as-prepared PAA/betaine elastomers with different water contents.

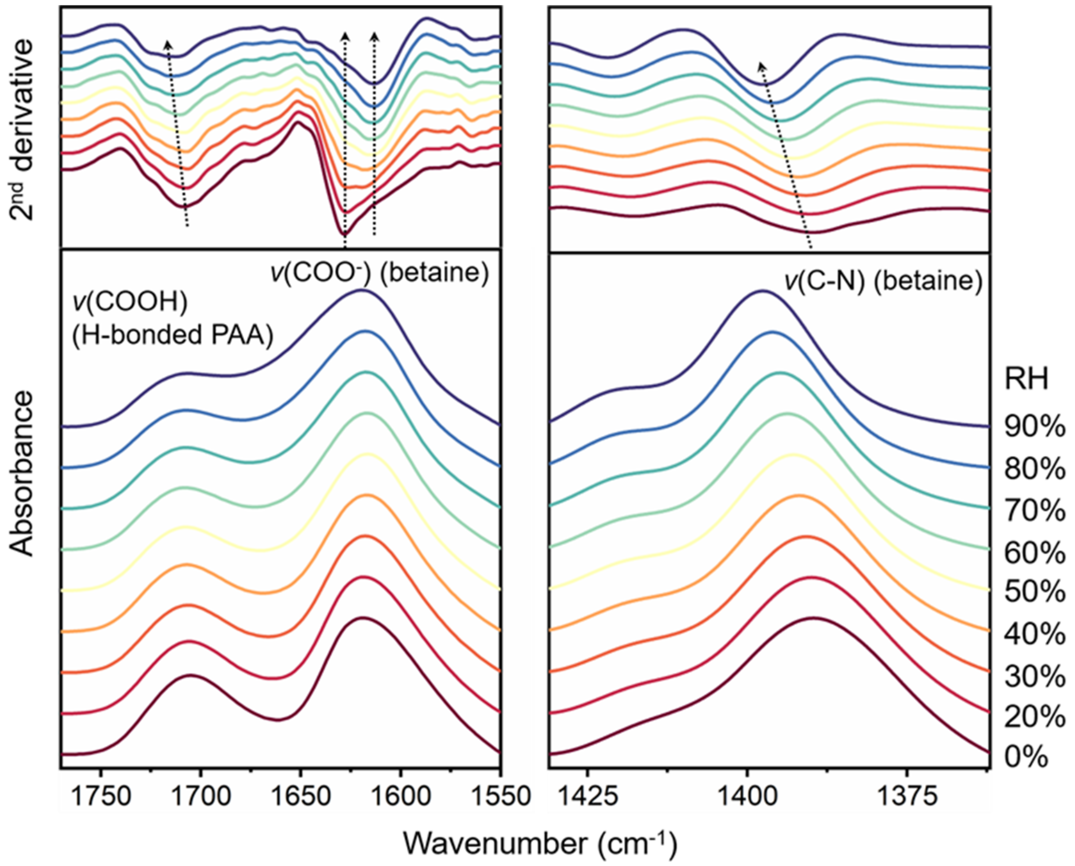

Supplementary Fig. 16. ATR-FTIR and corresponding second derivative spectra of PAA/betaine elastomer equilibrated at different humidities. With the increase of humidity, *ν*(COOH) (H-bonded PAA) and *ν*(C-N) (betaine) both shift to higher wavenumbers, indicating the water-induced weakening of PAA dimeric H-bonds and betaine ionic complexes. Moreover, judging from the second derivative spectra of *ν*(COO^-), a binary change is observed indicating the conversion of ionic complexed COO^- at 1633 cm^{-1} to hydrated COO^- at 1627 cm^{-1} at higher humidities.

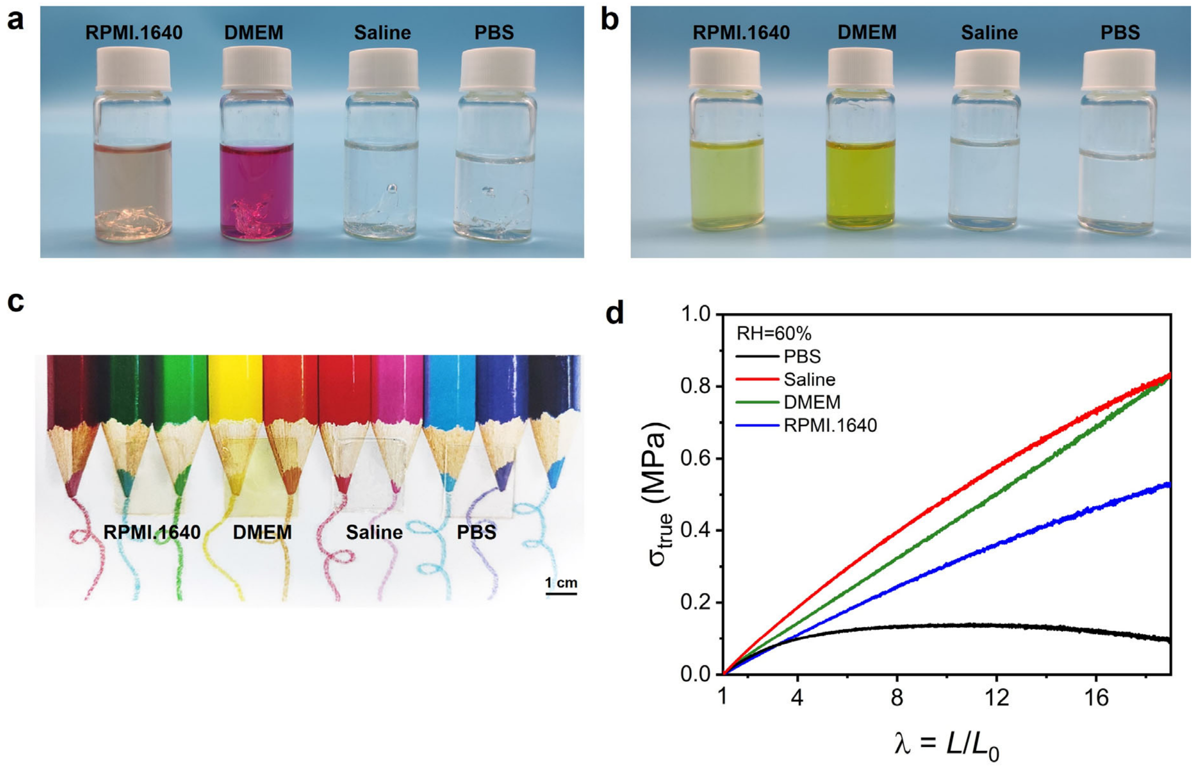

Supplementary Fig. 17. Recyclability of PAA/betaine elastomer in biologically relevant media. Photos of PAA/betaine elastomer **a**) before and **b**) after dissolving in RPMI.1640, and DMEM (high glucose) culture media, normal saline, and PBS buffer solutions, respectively. **c** Photo of the recasted PAA/betaine elastomer films. **d** Tensile curves of the recasted films at RH 60%. All the films are transparent and highly stretchable. However, since the abundant ions in the biologically relevant media strongly interrupt the physical crosslinks of PAA/betaine elastomer, strain-softening behavior is observed for all the four samples.

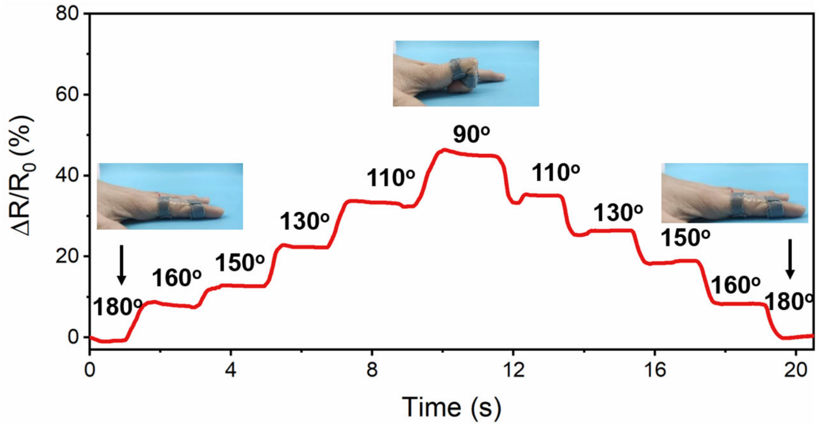

Supplementary Fig. 18. The recorded resistance variations of PAA/betaine elastomer adhered on human finger at different bending angles.

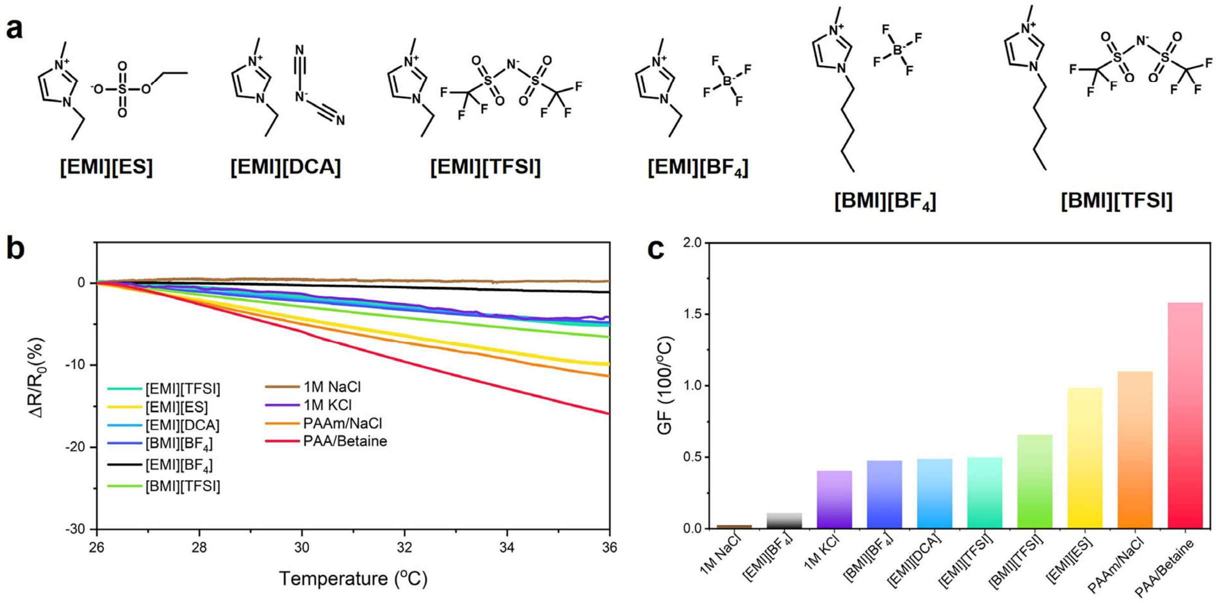

Supplementary Fig. 19. Temperature-dependent resistance variations and corresponding gauge factors of ionic liquids, saline solutions, PAAm/NaCl hydrogel and PAA/betaine elastomer. **a** Chemical structures of ionic liquids ([EMI][ES], [EMI][DCA], [EMI][TFSI], [EMI][BF_4], [BMI][BF_4], and [BMI][TFSI]). **b** Temperature-dependent resistance variations ($\Delta R/R_0$) and **c** corresponding gauge factors of ionic liquids, saline solutions (1 M KCl, 1 M NaCl), PAAm/NaCl hydrogel and PAA/betaine elastomer.

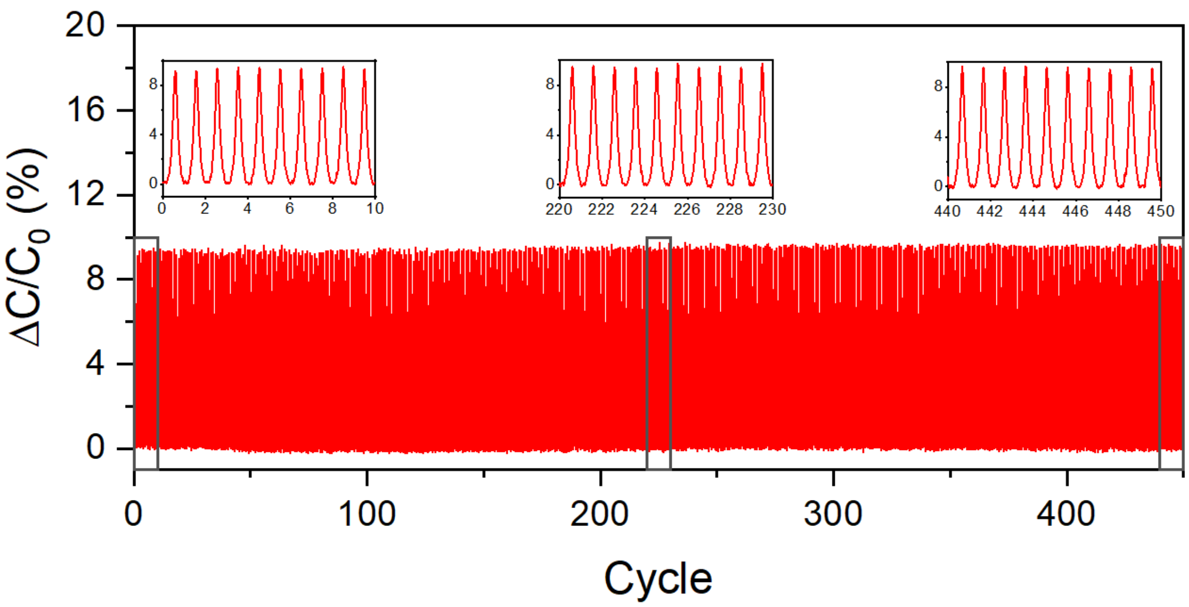

Supplementary Fig. 20. Capacitance response curve of PAA/betaine elastomer under the dynamically applied pressure of 2.45 kPa for 450 cycles.

Supplementary Table 1. Fitting parameters (τ* and β) in the simulation of iso-strain–stress relaxation results (Fig. 2d).

Temperature/^{o}C	τ^*	β
20	353.35	0.438
30	128.53	0.514
40	63.77	0.543
50	36.21	0.530

Supplementary Table 2. Final results of the multiplication of the signs of each cross-peak in 2DCOS synchronous and asynchronous spectra of PAA/betaine elastomer (Fig. 2f).

1392	-	-	-	-	
1402	+	-	+		
1639	+	-			
1701	+				
1728					
	1728	1701	1639	1402	1392

According to Noda's rule, the final specific order for PAA/betaine elastomer during heating is given as follows: 1392 → 1728 → 1639 → 1402 → 1701 cm^{-1} (→ means prior to or earlier than), i.e. *v*(C-N) (betaine-betaine complex) → *v*(COOH) (free PAA) → *v*(COO^-) (betaine) → *v*(C-N) (betaine-PAA complex) → *v*(C=O) (H-bonded PAA).

Supplementary Table 3. Extended comparison of the overall performance between this work and previously reported polyzwitterion-based hydrogels and ionic skin materials.

Ionic skin materials	Mechano response	Self-healing	Max. strain (%)	Elastic recovery ratio (%)	Transparency (%)	Air stability	Anti-freezing (ºC)	Adhesion	Ref.
PMAEDS/CNF hydrogel	Softening	None	920	N.A.	High	Poor	N.A.	Yes	[S6]
PDMAPS/silk fibroin/$CaCl_2$ hydrogel	Softening	24 h	6000	N.A.	High	Good	N.A.	Yes	[S7]
PAA-co-DMAPS/NaCl hydrogel	Softening	2 h, 100%	>10000	N.A.	90	Poor	N.A.	Yes	[S8]
PDA-clay-PSBMA hydrogel	Softening	24 h, 80%	900	93.8	Low	Poor	N.A.	Yes	[S9]
pCB/pSB ZEN hydrogel	Softening	None	169.9	N.A.	High	Poor	N.A.	N.A.	[S10]
Dopamine-triggered PSBMA hydrogel	N.A.	Fast	>350	N.A.	~90	N.A.	N.A.	Yes	[S11]
PAAm/PF127 hydrogel	Softening	Yes	2716	~95	83.8	Poor	N.A.	N.A.	[S12]
HPC/PVA DN hydrogel	Softening	None	975	~90	Opaque	Poor	N.A.	N.A.	[S13]
PAA-MEA organohydrogel	Softening	None	868	~100	~100	Good	-90	N.A.	[S14]
PVA-CNF organohydrogel	Softening	None	660	Good	90	Good	-70	N.A.	[S15]
Cellulose-IL dynamic gel	Softening	15 min, ~100%	~105	N.A.	>90	Good	N.A.	Yes	[S16]
PolyTA ionogel	Softening	6 h, ~100%	>10000	99%	95	Good	N.A.	Yes	[S17]
PEA DN ionogel	Softening	None	>5000	~94	95	Good	-70	Yes	[S18]
PVDF-HFP ionogel	Softening	24 h, 99.1%	~2000	~80	98	Good	N.A.	N.A.	[S19]
PIL/TFSI elastomer	Softening	2 h, ~100	540	95	Opaque	Good	N.A.	Yes	[S20]
PAAm-IL gel	Softening	Yes	900	N.A.	92	N.A.	-20	N.A.	[S21]
PAAm/ChCl elastomer	Softening	72 h, 94	450	N.A.	95	Good	-23	N.A.	[S22]
PAA/betaine elastomer	Stiffening	12 h，100%	1600	97.9	99.7	Good	-40	Yes	This work

Note: PMAEDS = PDMAPS = PSBMA. None of self-healing property refers to chemically or nanocrystal-crosslinked gels that are considered to be unable to heal themselves once damaged. Air stability means the stability of the material for maintaining stable ionic conduction as exposed in air. For gels, air stability is closed related to their ability of solvent retention in air.

Supplementary references

S1. Sun, J.-Y., Keplinger, C., Whitesides, G. M., Suo, Z. Ionic skin. *Adv. Mater.* **26**, 7608-7614 (2014).

S2. Noda, I. Two-dimensional infrared spectroscopy. *J. Am. Chem. Soc.* **111**, 8116-8118 (1989).

S3. Noda, I. Two-dimensional infrared (2D IR) spectroscopy: Theory and applications. *Appl. Spectrosc.* **44**, 550-561 (1990).

S4. Sun, S.-T., Wu, P.-Y. Spectral insights into microdynamics of thermoresponsive polymers from the perspective of two-dimensional correlation spectroscopy. *Chin. J. Polym. Sci.* **35**, 700-712 (2017).

S5. Noda, I., Ozaki, Y. Two-dimensional correlation spectroscopy: Applications in vibrational and optical spectroscopy. (John Wiley & Sons, Hoboken, 2005).

S6. Mo, F., et al. Zwitterionic sulfobetaine hydrogel electrolyte building separated positive/negative ion migration channels for aqueous Zn-MnO_2 batteries with superior rate capabilities. *Adv. Energy Mater.* **10**, 2000035 (2020).

S7. Lei, Z., Zhu, W., Zhang, X., Wang, X., Wu, P. Bio-inspired ionic skin for theranostics. *Adv. Funct. Mater.* **30**, 2008020 (2020).

S8. Lei, Z., Wu, P. A supramolecular biomimetic skin combining a wide spectrum of mechanical properties and multiple sensory capabilities. *Nat. Commun.* **9**, 1134 (2018).

S9. Pei, X., Zhang, H., Zhou, Y., Zhou, L., Fu, J. Stretchable, self-healing and tissue-adhesive zwitterionic hydrogels as strain sensors for wireless monitoring of organ motions. *Mater. Horiz.* **7**, 1872-1882 (2020).

S10. Dong, D., et al. High-strength and fibrous capsule–resistant zwitterionic elastomers. *Sci. Adv.* **7**, eabc5442 (2021).

S11. Zhang, C., Zhou, Y., Han, H., Zheng, H., Xu, W., Wang, Z. Dopamine-triggered hydrogels with high transparency, self-adhesion, and thermoresponse as skinlike sensors. *ACS Nano* **15**, 1785-1794 (2021).

S12. Li, Y., et al. Ultra-stretchable, variable modulus, shape memory multi-purpose low hysteresis hydrogel derived from solvent-induced dynamic micelle sea-island structure. *Adv. Funct. Mater.* **31**, 2011259 (2021).

S13. Zhou, Y., et al. Highly stretchable, elastic, and ionic conductive hydrogel for artificial soft electronics. *Adv. Funct. Mater.* **29**, 1806220 (2019).

S14. Liu, J., et al. Ionic conductive organohydrogels with dynamic pattern behavior and multi-environmental stability. *Adv. Funct. Mater.* **31**, 2101464 (2021).

S15. Ye, Y., Zhang, Y., Chen, Y., Han, X., Jiang, F. Cellulose nanofibrils enhanced, strong,

stretchable, freezing-tolerant ionic conductive organohydrogel for multi-functional sensors. *Adv. Funct. Mater.* **30**, 2003430 (2020).

S16. Zhao, D., et al. A dynamic gel with reversible and tunable topological networks and performances. *Matter* **2**, 390-403 (2020).

S17. Wang, Y., Sun, S., Wu, P. Adaptive ionogel paint from room-temperature autonomous polymerization of α-thioctic acid for stretchable and healable electronics. *Adv. Funct. Mater.* **31**, 2101494 (2021).

S18. Cao, Z., Liu, H., Jiang, L. Transparent, mechanically robust, and ultrastable ionogels enabled by hydrogen bonding between elastomers and ionic liquids. *Mater. Horiz.* **7**, 912-918 (2020).

S19. Cao, Y., et al. Self-healing electronic skins for aquatic environments. *Nat. Electron.* **2**, 75-82 (2019).

S20. Qu, X., et al. Solid-state and liquid-free elastomeric ionic conductors with autonomous self-healing ability. *Mater. Horiz.* **7**, 2994-3004 (2020).

S21. Liu, Z., et al. Poly(ionic liquid) hydrogel-based anti-freezing ionic skin for a soft robotic gripper. *Mater. Horiz.* **7**, 919-927 (2020).

S22. Li, R. a., et al. Autonomous self-healing, antifreezing, and transparent conductive elastomers. *Chem. Mater.* **32**, 874-881 (2020).
