# Supplementary material for: Skin-like mechanoresponsive self-healing ionic elastomer from supramolecular zwitterionic network
Source: Nat Commun. 2021 Jul 2;12:4082. doi: 10.1038/s41467-021-24382-4 (PMC8253733; doi:10.1038/s41467-021-24382-4)
Supplement: Supplementary file 2 — Description of Additional Supplementary Files [file 41467_2021_24382_MOESM2_ESM.pdf]

Description of Additional Supplementary Files

File Name: Supplementary Movie 1
Description: High elasticity, mechanical compliance, and adhesion of PAA/betaine elastomer

File Name: Supplementary Movie 2
Description: Self-healing of PAA/betaine elastomer
